# RuCo/ZrO_2_ Tandem Catalysts with Photothermal Confinement Effect for Enhanced CO_2_ Methanation

**DOI:** 10.1002/advs.202406828

**Published:** 2024-07-10

**Authors:** Fan Yang, Xiaoyu Liu, Chuanshun Xing, Zizheng Chen, Lili Zhao, Xingwu Liu, Wenqiang Gao, Luyi Zhu, Hong Liu, Weijia Zhou

**Affiliations:** ^1^ Institute for Advanced Interdisciplinary Research (iAIR) School of Chemistry and Chemical Engineering University of Jinan Jinan 250022 P. R. China; ^2^ State Key Laboratory of Crystal Materials Shandong University Jinan 250100 P. R. China; ^3^ Synfuels China Technology Co. Ltd. Leyuan Second South Street Yanqi Development Zone Huairou Beijing 101407 P. R. China

**Keywords:** bimetallic nanoparticles, CO_2_ methanation, photothermal confinement effect, tandem catalysts

## Abstract

Photothermal CO_2_ methanation reaction represents a promising strategy for addressing CO_2_‐related environmental issues. The presence of efficient tandem catalytic sites with a localized high‐temperature is an effective pathway to enhance the performance of CO_2_ methanation. Here the bimetallic RuCo nanoparticles anchored on ZrO_2_ fiber cotton (RuCo/ZrO_2_) as a photothermal catalyst for CO_2_ methanation are prepared. A significant photothermal CO_2_ methanation performance with optimal CH_4_ selectivity (99%) and rate (169.93 mmol g_cat_^−1^ h^−1^) is achieved. The photothermal energy of the RuCo bimetallic nanoparticles, confined by the infrared insulation and low thermal conductivity of the ZrO_2_ fiber cotton (ZrO_2_ FC), provides a localized high‐temperature. In situ spectroscopic experiments on RuCo/ZrO_2_, Ru/ZrO_2_, and Co/ZrO_2_ indicate that the construction of tandem catalytic sites, where the Co site favors CO_2_ conversion to CO while incorporating Ru enhances CO^*^ adsorption for subsequent hydrogenation, results in a higher selectivity toward CH_4_. This work opens a new insight into designing tandem catalysts with a photothermal confinement effect in CO_2_ methanation reaction.

## Graphical abstract

The bimetallic RuCo nanoparticles anchored on ZrO_2_ fiber cotton (RuCo/ZrO_2_) as a photothermal catalyst for CO_2_ methanation. The RuCo/ZrO_2_ realizes a superior localized high‐temperature by photothermal confinement effect. The construction of Co–Ru tandem catalytic sites is able to promote the conversion of CO_2_ to CH_4_ in photothermal CO_2_ methanation.

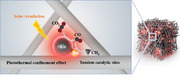

## 1 Introduction

Given the urgent challenges posed by climate change and environmental issues, it is crucial to transition from relying on fossil fuels to embracing renewable and carbon‐neutral energy technologies. Particularly, the conversion of carbon dioxide (CO_2_) into liquid and gaseous fuels through renewable energy represents a promising energy storage strategy that allows for simultaneous decarbonization and substitution of fossil fuels.^[^ ^1^ ^]^ Methane (CH_4_) stands out as one of the preferred fuel options due to its high energy density and available technologies for back‐end applications. Thus, solar‐driven photothermal CO_2_ methanation presents an effective pathway for producing high‐quality solar fuels.^[^ ^2^ ^]^

A prerequisite for achieving efficient solar‐driven photothermal CO_2_ methanation is the generation of a localized high‐temperature field driven by solar energy, which heavily depends on the inherent properties and structural characteristics of the photothermal materials.^[^ ^3^ ^]^ Group VIII metals have demonstrated outstanding light absorption and conversion properties from light to heat,^[^ ^4^ ^]^ however, the main limitation in achieving optimal photothermal temperature is the rapid dissipation of heat that occurs on the surface of the material.^[^ ^5^ ^]^ It is vital to minimize heat dissipation by reducing both infrared radiation (IR) and heat conduction losses to achieve a localized high‐temperature. In previous strategies, catalysts that combined with metals and core–shell structure,^[^ ^6^ ^]^ selective light absorber,^[^ ^7^ ^]^ and infrared insulating materials^[^ ^8^ ^]^ were widely employed. Zirconium oxide fiber cotton (ZrO_2_ FC) has gained popularity as an infrared insulation material with low thermal conductivity (0.168–0.212 W m^−1^⋅K) when filled with air.^[^ ^9^ ^]^ This makes it an ideal candidate as a support material for photothermal catalysts because it simultaneously enhances light absorption and minimizes heat dissipation, offering a feasible approach to achieve a localized high‐temperature.

The photothermal CO_2_ methanation reaction is widely believed to occur via two reaction steps, that is CO_2_‐to‐CO and CO‐to‐CH_4_.^[^ ^10^ ^]^ The formation and conversion of CO intermediates (CO^*^) on catalysts play a key role in the CO_2_ methanation reaction.^[^ ^11^ ^]^ Therefore, optimizing the adsorption of reaction intermediates on the catalytic site is essential. Non‐precious metal Co‐based catalysts have been widely reported for economically viable CO_2_ methanation.^[^ ^12^ ^]^ However, the conversion of CO is more favorable compared to the relatively inert conversion of CH_4_ due to the weak adsorption energy of CO on Co‐based catalysts,^[^ ^13^ ^]^ which limits its further hydrogenation. Noble metal Ru‐based catalysts are conducive to CO adsorption, which ensures the subsequent hydrogenation reaction.^[^ ^14^ ^]^ Thus, incorporating Ru into the Co‐based catalyst to construct tandem catalytic sites for simultaneous regulating the two‐step, i.e., CO_2_‐to‐CO and CO‐to‐CH_4_, is a valid approach to achieve an optimal CH_4_ selectivity in the CO_2_ methanation reaction.

In this work, we present a catalyst design involving RuCo bimetallic nanoparticles anchored on ZrO_2_ FC (RuCo/ZrO_2_), which achieved a high rate (169.93 mmol g_cat_^−1^ h^−1^) and selectivity (99%) for the CO_2_ methanation reaction. We demonstrated that the RuCo/ZrO_2_ catalyst exhibits a photothermal confinement effect, resulting in a localized high‐temperature (323.7 °C at 1.83 W cm^−2^), which effectively drives the CO_2_ methanation reaction. Combined with in situ diffuse reflectance infrared Fourier transform spectroscopy (in situ DRIFTS) and the density functional theory (DFT) calculations investigations, we have demonstrated that the construction of CoRu tandem catalytic sites synchronously regulates the conversion of CO_2_‐to‐CO and CO‐to‐CH_4_, thereby achieving a high selectivity for CH_4_.

## 2 Results and Discussion

### 2.1 Characterization of Photothermal Catalysts

**Figure 1:**
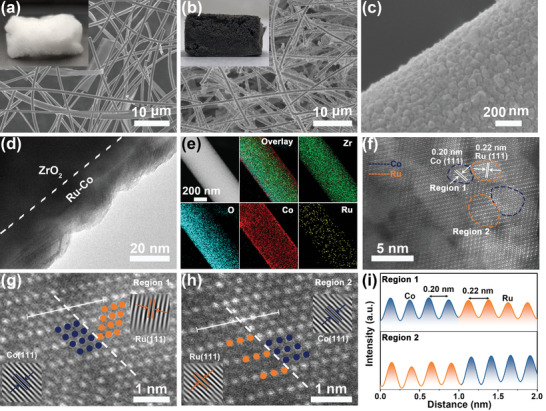
SEM images of a) ZrO_2_ FC and b,c) RuCo/ZrO_2_, the insets show the optical pictures. d) TEM image of RuCo/ZrO_2_. e) EDX mapping and f) HAADF–STEM images of RuCo/ZrO_2_. g,h) Enlarged HAADF–STEM images of the marked regions. i) Intensity profiles measured from HAADF–STEM image of Co (111) and Ru (111) for RuCo/ZrO_2_.

The RuCo/ZrO_2_ photothermal catalyst was synthesized using a hydrothermal method with ZrO_2_ FC as the support. The field emission scanning electron microscope (SEM) image of ZrO_2_ FC exhibited a 3D network structure composed of smooth fibers (**Figure** **1**a). The optical pictures of ZrO_2_ FC showed a white and aerogel‐like morphology (inset of Figure 1a). The color transformed from white to black after anchoring RuCo bimetallic nanoparticles on ZrO_2_ FC (inset of Figure 1b). SEM images showed that the surface roughness of RuCo/ZrO_2_ had a noticeable increase compared to ZrO_2_ FC (Figure 1b; Figures S1 and S2, Supporting Information). SEM and the transmission electron microscopy (TEM) images of RuCo/ZrO_2_ showed the uniform distribution of RuCo nanoparticles on the surface of ZrO_2_ FC, forming a relatively textured surface (Figure 1c,d). The distribution of nanoparticles size were analyzed, and the average particle size was calculated to be 39.84 nm (Figure S3, Supporting Information). The XRD pattern of RuCo/ZrO_2_ confirmed the formation of metallic Co, i.e., the peak at 44.23° attributed to the (111) plane of Co (PDF#89‐7093) (Figure S4, Supporting Information). However, the absence of Ru metal or alloy phase could be attributed to a small amount of Ru, which was confirmed to be 0.15 wt% by inductively coupled plasma atom emission spectrometry (ICP) analysis (Table S1, Supporting Information). The corresponding energy‐dispersive X‐ray spectroscopy (EDX) mapping images of RuCo/ZrO_2_ and line‐scanning EDX analysis showed a uniform distribution of Co and Ru on the surface of ZrO_2_ FC (Figure 1e; Figure S5, Supporting Information), suggesting the successful construction of RuCo/ZrO_2_. Spherical aberration‐corrected high‐angle annular dark‐field–scanning transmission electron microscopy (HAADF–STEM) images suggested adjacent Co (blue) and Ru (orange) nanoparticle fragments on the RuCo/ZrO_2_ catalyst. (Figure 1f). The fast Fourier transform (FFT) and fast Fourier inverse transform (IFFT) patterns of the marked regions verified the presence of Co (111) and Ru (111) facets (Figure S6, Supporting Information). The entire lattice showed highly ordered arrays of atoms observed by the enlarged HAADF–STEM images of the marked regions (Figure 1g,h). The Co (111) and Ru (111) directions were labeled based on the orientations of their respective unit cells, while the intensity profile displayed a periodic oscillation pattern in both directions (Figure 1i). The HAADF–STEM images of RuCo/ZrO_2_ confirmed the existence of bimetallic RuCo interface.

**Figure 2:**
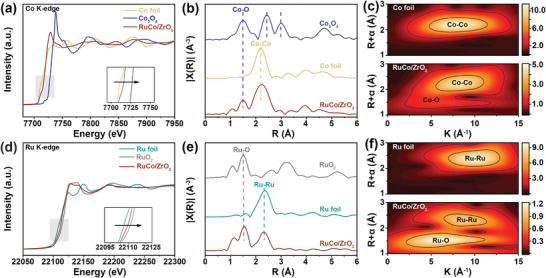
a) Normalized Co K‐edge and d) Ru K‐edge XANES of RuCo/ZrO_2_. The FT‐EXAFS spectra derived from EXAFS of b) Co K‐edge and e) Ru K‐edge. Wavelet transforms of c) Co K‐edge and f) Ru K‐edge EXAFS signals of RuCo/ZrO_2_.

The valence state and local coordination environment of RuCo/ZrO_2_ were determined using X‐ray absorption spectroscopy (XAS). The adsorption edges of both Co K‐edge and Ru K‐edge showed a shift toward a higher energy direction, indicating an increase in their valence state monitored by the X‐ray absorption near‐edge structure (XANES) spectra (**Figure** **2**a,d). The extended X‐ray absorption fine structure (EXAFS) spectra suggested bond lengths of ≈2.20 and 2.40 Å for Co–Co and Ru–Ru bonds, respectively (Figure 2b,e), indicating that the Co and Ru species predominantly exist in metallic form for RuCo/ZrO_2_. The observed Co–O and Ru–O bonds confirmed a strong interaction between RuCo bimetallic nanoparticles and ZrO_2_ that leads to their bonding with each other.^[^ ^15^ ^]^ The coordination of Co with Ru in both k and R space was demonstrated by wavelet transform extended X‐ray absorption fine structure (WT‐EXAFS) analysis (Figure 2c,f). Similar to the FT‐EXAFS spectra, the WT signal related to the Co–Co and Ru–Ru bonds as well as Co–O and Ru–O bonds were detected in RuCo/ZrO_2_. It has been confirmed that Co and Ru species mainly existed in bimetallic nanoparticles form and bonded with ZrO_2_ through Ru–O and Co–O interactions. The evidence of RuCo interaction with ZrO_2_ is further supported by the observed shifts in both Zr^4+^ 3d and Co^0^ 2p binding energies in the X‐ray photoelectron spectroscopy (XPS) spectra of ZrO_2_, RuCo, and RuCo/ZrO_2_ samples. (Figure S7, Supporting Information). The absence of Ru peaks in XPS could be attributed to a small amount of Ru (0.15 wt% by ICP, Figure S8, Supporting Information). Therefore, the RuCo bimetallic nanoparticles were successfully anchored on ZrO_2_ by integrating the TEM, HAADF–STEM, XAS, and XPS results.

### 2.2 Photothermal Performance of Catalysts

**Figure 3:**
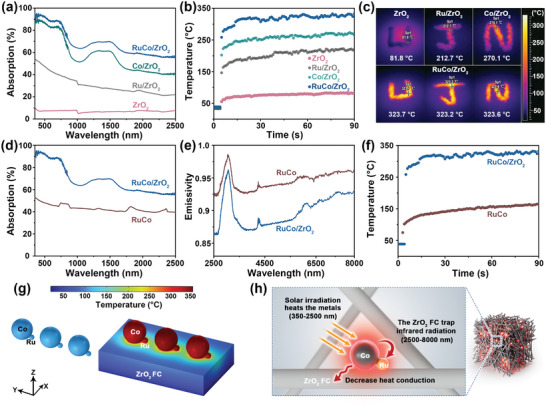
a) UV‐Vis‐NIR absorption spectra, b) surface temperature profiles, and c) the infrared thermal images of ZrO_2_, Ru/ZrO_2_, Co/ZrO_2_, and RuCo/ZrO_2_ under the 1.83 W cm^−2^ irradiation. d) The UV–vis–NIR absorption spectrum, e) IR emissivity curve, and f) surface temperature profiles of RuCo and RuCo/ZrO_2_. g) The simulation of temperature distributions for RuCo and RuCo/ZrO_2_ under light irradiation. h) The schematic diagram of the photothermal confinement effect for RuCo/ZrO_2_.

The capability of light absorption and photothermal conversion of RuCo/ZrO_2_ were investigated by comparing the single metal‐loaded ZrO_2_, i.e., Co/ZrO_2_ and Ru/ZrO_2_, as well as bimetallic RuCo nanoparticles without ZrO_2_, i.e., RuCo. The characterization of the control materials is shown in Figure S9 (Supporting Information). The light absorption capability of Ru/ZrO_2_, Co/ZrO_2_, RuCo/ZrO_2_, and ZrO_2_ were determined by the ultraviolet‐visible‐near infrared (UV–vis–NIR) absorption spectra (**Figure** **3**a; Figure S10, Supporting Information). The RuCo/ZrO_2_ showed an enhanced light absorption compared to ZrO_2_ and Ru/ZrO_2_ range from UV to NIR wavelengths. The similar absorption peaks between RuCo/ZrO_2_ and Co/ZrO_2_ suggested that metal cobalt is used as a light‐absorbing material in RuCo/ZrO_2_. The surface temperature of RuCo/ZrO_2_ increased substantially within 90 s and stabilized at 323.7 °C as determined by an infrared camera (Figure 3b,c), which was higher than the temperatures of ZrO_2_ (81.8 °C), Ru/ZrO_2_ (212.7 °C), and Co/ZrO_2_ (270.1 °C) under the 1.83 W cm^−2^ irradiation, respectively. The accuracy of temperature measurement was confirmed by a thermocouple obtaining consistent results from an infrared camera (Tables S2 and S3, Supporting Information). This observation indicated that the RuCo/ZrO_2_ showed optimal photothermal conversion capability.

The photothermal confinement effect of ZrO_2_ FC was demonstrated by the light absorption and IR emissivity curves of RuCo without ZrO_2_ FC supports and RuCo/ZrO_2_. RuCo was anchored on the ZrO_2_ FC with a 3D network structure, which effectively enhances light scattering and thus improves light absorption in the UV–vis–NIR spectral regions (350–2500 nm) compared to RuCo (Figure 3d). The emissivity of RuCo/ZrO_2_ is lower than that of RuCo within the wavelength range of 2500–8000 nm, suggesting a lower intensity of infrared radiation (Figure 3e). A low thermal conductivity of ZrO_2_ FC, i.e., 0.044 W (m⋅K)^−1^ @ 330 °C, ensured the ability to effectively minimize heat conduction loss. Furthermore, the difference in photothermal temperatures, i.e., RuCo/ZrO_2_ (323.7 °C) and RuCo (164.1 °C), indicated that ZrO_2_ as a support can reduce heat dissipation by photothermal confinement effect (Figure 3f). The utilization of ZrO_2_ FC with photothermal confinement effect as a catalyst support represents a significant pathway to realize a localized high‐temperature on the surface of catalyst, as evidenced by the measurement of photothermal temperatures of different metal oxide powder samples loaded with RuCo (Figures S11–S14, Supporting Information).

The photothermal confinement effect of RuCo/ZrO_2_ was demonstrated by simulating the distributions of induced electric field and temperatures on RuCo/ZrO_2_ and RuCo using finite element methods (Figure 3g; Figure S15, Supporting Information). The electric field of RuCo was predominantly generated at the surface of Co and Ru nanoparticles under light irradiation, reaching its maximum at the interface.^[^ ^16^ ^]^ The temperature distribution demonstrated that photothermal energy rapidly diffuses from RuCo nanoparticles to the surrounding medium, resulting in a lower photothermal temperature (Figure S15, Supporting Information). An enhanced electric field of RuCo/ZrO_2_ was distributed on the interface between RuCo nanoparticles and ZrO_2_ FC. The decay process of the enhanced localized electromagnetic field would drive the produced heat arising,^[^ ^17^ ^]^ resulting in a temperature increase around the catalytic sites. As a result, a localized high‐temperature field (Figure 3g) was achieved, which guaranteed the activation of the reactant molecules through electron acceptance in their antibonding orbitals.^[^ ^18^ ^]^ The schematic diagram illustrated the photothermal confinement effect for RuCo/ZrO_2_ (Figure 3h). The presence of RuCo nanoparticles serves as hotspots by light absorption (350–2500 nm) and photothermal conversion. The ZrO_2_ FC acts as an insulation layer, which was able to trap the infrared radiation emitted from the “hot” RuCo nanoparticles (2500–8000 nm). Additionally, the low thermal conductivity of ZrO_2_ FC can reduce heat conduction loss. Therefore, the RuCo/ZrO_2_ realized a superior localized high‐temperature by photothermal confinement effect to minimize heat dissipation, which can efficiently. drive photothermal CO_2_ methanation.

### 2.3 Photothermal Catalytic Performance of Catalysts

**Figure 4:**
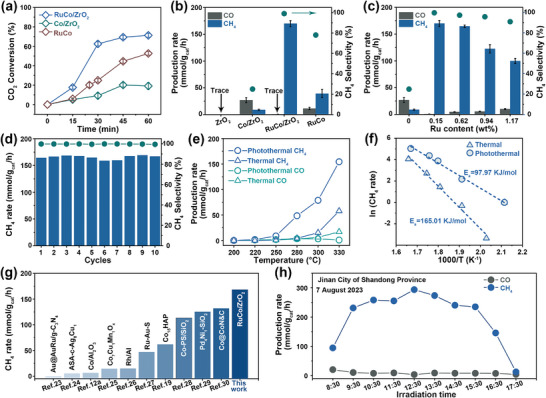
a) CO_2_ conversion of Co/ZrO_2_, RuCo/ZrO_2_ and RuCo. b) The production rate of ZrO_2_, Co/ZrO_2_, RuCo/ZrO_2_ and RuCo. c) The relationship between production rate and Ru loading amount. d) Cycle stability test of the RuCo/ZrO_2_, (Testing conditions: CO_2_/H_2_ = 1:4, initial reactor pressure = 0.1 MPa, light intensity = 1.83 W cm^−2^). e) The production rate and f) the Arrhenius plot with activation energies of RuCo/ZrO_2_ in the process of photothermal and thermal catalytic reaction. g) The comparison of photothermal catalytic activity with other reported catalysts. h) The production rate of the RuCo/ZrO_2_ as a function of time under ambient sunlight irradiation.

The CO_2_ conversion rate and yield plots in 60 min of various catalysts, including Co/ZrO_2_, RuCo, and RuCo/ZrO_2_ were evaluated (**Figure** **4**a; Figure S16, Supporting Information). The RuCo/ZrO_2_ exhibited an optimal CO_2_ conversion rate of 60% in 30 min, surpassing both Co/ZrO_2_ (9%) and RuCo (25%). However, the CO_2_ conversion rate gradually decelerated due to reaching catalytic equilibrium in the closed batch system when beyond 45 min.^[^ ^19^ ^]^ The photothermal catalytic performance of various catalysts were evaluated in the closed batch reactor at atmospheric pressure (Figure 4b). The ZrO_2_ exhibited negligible catalytic activity while the Co/ZrO_2_ catalyst showed an improved selectivity of 77% for CO (33.04 mmol g_cat_^−1^ h^−1^). This observation suggested that the Co site has a strong preference for converting CO_2_ into CO, which was consistent with previous reports.^[^ ^13^, ^20^ ^]^ The incorporation of a small amount of Ru (0.15 wt%) tuned the CH_4_ selectivity from 23% on Co/ZrO_2_ to an impressive 99% and improved the CH_4_ rate to 169.93 mmol g_cat_^−1^ h^−1^ on RuCo/ZrO_2_. We also compared the selectivities of the different catalysts at the same conversion to exclude the influence of thermodynamic equilibrium on the analysis of the results (Figure S17, Supporting Information). This result indicated that incorporating Ru enhances the efficiency of converting CO_2_ into CH_4_. However, the Ru/ZrO_2_ catalysts with varying amounts of Ru (0.15, 0.61, 0.94, 1.17 wt%) exhibited relatively low catalytic activities for CH_4_ (Figure S18, Supporting Information), suggesting that the construction of RuCo bimetallic tandem catalytic sites is essential for the realizing CO_2_ methanation reaction on RuCo/ZrO_2_. However, the RuCo without ZrO_2_ FC exhibited a decreased CH_4_ selectivity (78%) and rate (39.18 mol g_cat_^−1^ h^−1^) under the same illumination condition, suggesting that the uniformly dispersed RuCo nanoparticles on ZrO_2_ FC with photothermal confinement effect were conducive to realize the high catalytic activity. The photothermal CO_2_ methanation activity of RuCo/ZrO_2_ catalysts with varying amounts of Ru (0, 0.15, 0.62, 0.94, 1.17 wt%, Table S1, Supporting Information) was depicted in Figure 4c. The CH_4_ selectivity and rate decrease as the Ru content increases. This can be rationalized by the fact that the introduction of excessive Ru resulted in the presence of larger‐sized Ru nanoparticles and lower catalytic activity (Figures S19–S21, Supporting Information), similar observations have been reported.^[^ ^21^ ^]^ Thus, the RuCo/ZrO_2_‐0.15 wt% was selected as the optimal catalyst, denoted as RuCo/ZrO_2_. It should be noted that the physical mixture of RuCo nanoparticles and ZrO_2_ FC (M‐RuCo/ZrO_2_) did not show a high activation for CO_2_ methanation (Figure S22, Supporting Information), indicating that the strong interaction between RuCo nanoparticles and ZrO_2_ FC favors catalytic performance. The reinforced catalytic stability of the RuCo/ZrO_2_ was demonstrated through 10 consecutive cycles of testing (Figure 4d; Figure S23, Supporting Information). The structural stability of the RuCo/ZrO_2_ was confirmed by observing catalyst characterization after the photothermal catalytic reaction (Figures S24–S27, Supporting Information). In contrast, limited stability was observed over 5 consecutive cycles for the RuCo nanoparticles (Figures S28 and S29, Supporting Information), indicating the essentiality of ZrO_2_ FC support as a guarantor of homogeneous dispersion of RuCo bimetallic nanoparticles and thus achieving catalytic stability.

The photothermal CO_2_ methanation performance and photothermal temperature were investigated to specify the potential of the RuCo/ZrO_2_ catalyst under various light intensities. It was observed that both the photothermal temperature and CH_4_ rate of RuCo/ZrO_2_ were increased with light intensity (Figure S30a, Supporting Information), indicating that the reaction primarily relied on the photothermal effect. It is worth noting that the catalytic performance of Ru/ZrO_2_ remained unsatisfactory despite reaching photothermal temperatures similar to RuCo/ZrO_2_ (Figure S30b, Supporting Information). This observation suggested that the exceptional photothermal performance of RuCo/ZrO_2_ stems not only from its high photothermal temperature but also from its intrinsic catalytic activity. The catalytic performance of RuCo/ZrO_2_, driven by both photothermal and thermal catalysis within the temperature range of 200–330 °C, was evaluated to demonstrate the impact of light irradiation (Figure 4e). The CH_4_ rate reached 1.67 mmol g_cat_^−1^ h^−1^, while the CH_4_ generation was not observed in the thermal process at 220 °C. The results demonstrated that light irradiation effectively improved the activity of photothermal catalytic CO_2_ methanation.^[^ ^22^ ^]^ The Arrhenius plots of RuCo/ZrO_2_ were fitted for both the photothermal and thermal processes (Figure 4f). It was observed that the activation energy (E_a_) for the photothermal process (97.97 kJ mol^−1^) was lower compared to that of the thermal process (165.01 kJ mol^−1^). The lower Ea suggested a lower kinetic barrier associated with photothermal catalysis. The catalytic activity of recently reported photothermal catalysts was normalized based on catalyst mass to further assess the photothermal catalytic performance of RuCo/ZrO_2_ (Figure 4g; Table S5, Supporting Information). The catalytic activity of RuCo/ZrO_2_ catalyst outperformed those of other catalysts, e.g., Au@AuRu/g‐C_3_N_4_,^[^ ^23^ ^]^ ASA‐c‐Ag_8_Cu_1_,^[^ ^24^ ^]^ Co/Al_2_O_3_,^[^ ^12a^ ^]^ Co_7_Cu_1_Mn_1_O_x_,^[^ ^25^ ^]^ Rh/Al,^[^ ^26^ ^]^ Ru‐Au‐S,^[^ ^27^ ^]^ Co_15_HAP,^[^ ^20^ ^]^ Co‐PS@SiO_2_,^[^ ^28^ ^]^ Pd_4_Ni_1_‐SiO_2_,^[^ ^29^ ^]^ Co@CoN&C^[^ ^30^ ^]^ et.al. It implied that the RuCo/ZrO_2_ catalyst exhibited a promising potential for practical implementation.

The outdoor sunlight driving CO_2_ methanation of RuCo/ZrO_2_ was evaluated using a solar concentrator system that was equipped with a parabolic reflector capable of concentrating sunlight over an irradiation area of 1 m^2^ (Figure S31, Supporting Information). The RuCo/ZrO_2_ catalyst was placed onto the bottle of a quartz batch reactor, which was filled with a mixture of H_2_ and CO_2_ gases in a ratio of 4:1. The experiment was conducted from 8:30 to 17:30 under natural sunlight on 7 August 2023, under cloudy weather and an ambient temperature ranging from 22 to 31 °C during daytime in Jinan City, Shandong Province, China (Table S4, Supporting Information). The CH_4_ rate peaked at 12:30 reaching 293.82 mmol g_cat_^−1^ h^−1^ (Figure 4h). This performance test system demonstrated that RuCo/ZrO_2_ was able to utilize outdoor sunlight to drive the CO_2_ methanation reaction without additional energy input.

### 2.4 Investigation of the Photothermal Catalytic Mechanism

**Figure 5:**
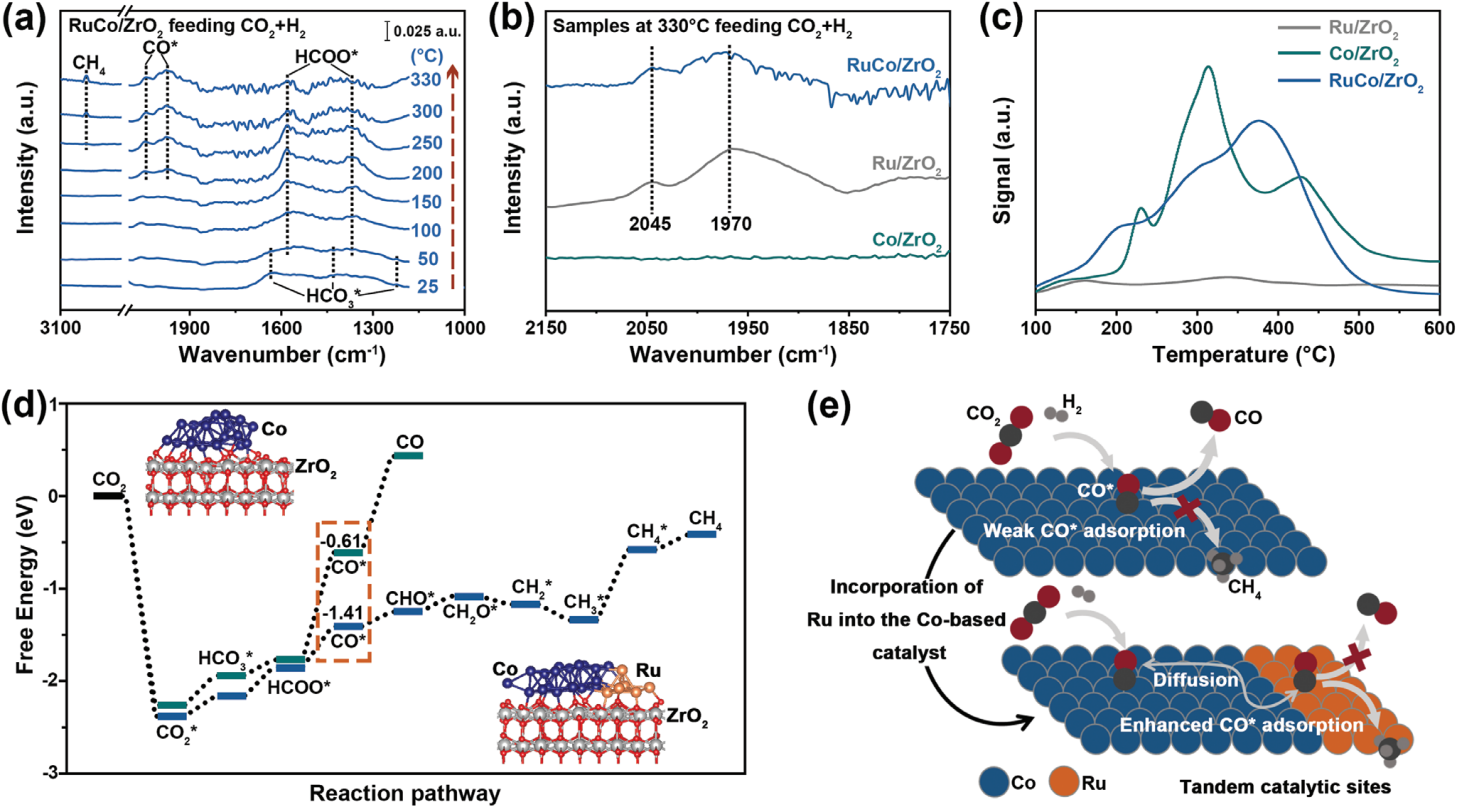
a) In situ DRIFTS during both light irradiation and temperature‐programmed CO_2_ methanation of a gas mixture containing the ratio of 1:4 with CO_2_/H_2_ on RuCo/ZrO_2_, and b) Ru/ZrO_2_, Co/ZrO_2_, and RuCo/ZrO_2_ at 330 °C in CO_2_ methanation. c) H_2_‐TPR comparison profiles of the Co/ZrO_2_ and RuCo/ZrO_2_. d) Free energy diagram of reaction pathways on Co/ZrO_2_ and RuCo/ZrO_2_. e) Schematic mechanism of photothermal CO_2_ methanation on Co/ZrO_2_ and RuCo/ZrO_2_.

The evolved intermediate species of CO_2_ methanation on Co/ZrO_2_, Ru/ZrO_2_, and RuCo/ZrO_2_ were determined by in situ DRIFTS under both light irradiation and temperature‐programmed (**Figure** **5**a; Figure S32, Supporting Information). The typical CO_2_ adsorption species, e.g., the bicarbonate^[^ ^31^ ^]^ (HCO_3_^*^) peaks at 1633, 1420, and 1222 cm^−1^ and formate^[^ ^32^ ^]^ (HCOO^*^) peaks at 1580 and 1369 cm^−1^, were detected in the low‐temperature range (Figure 5a). The HCOO* keep to increase substantially accompanied by the disappearance of HCO_3_^*^ species with the increasing temperature, indicating that the HCOO^*^ originated from the hydrogenation of HCO_3_^*^.^[^ ^33^ ^]^ The intensity of the HCOO^*^ peak increases and without generating subsequent hydrogenation products (CH_4_ or CO) when the temperature exceeds 150 °C on Co/ZrO_2_ (Figure S32a, Supporting Information), suggesting that the rapid desorption of adsorbed CO (CO^*^) from the catalyst surface inhibited its further hydrogenated to CH_4_.^[^ ^34^ ^]^ However, the intensity of the HCOO^*^ peak decreases accompanied by the appearance of linear‐CO^*^ (2052 cm^−1^)^[^ ^35^ ^]^ and bridge‐CO^*^ (1975 cm^−1^)^[^ ^36^ ^]^ on RuCo/ZrO_2_ as the temperature exceeds 250 °C (Figure 5a). A similar investigation demonstrated that the CO^*^ was produced by the dissociation of HCOO^*^.^[^ ^37^ ^]^ The peak of CH_4_ (3016 cm^−1^)^[^ ^38^ ^]^ appeared as the temperature increased to 250 °C, which demonstrated that the HCOO^*^ dissociation mediated CO^*^ hydrogenation pathway^[^ ^39^ ^]^ occurred on RuCo/ZrO_2_. The CO^*^ band was determined on RuCo/ZrO_2_ during CO_2_ methanation, indicating that the CO^*^ is a key intermediate for further hydrogenation to form CH_4_. The CO_2_ methanation process on Ru/ZrO_2_ and Co/ZrO_2_ were monitored to identify the adsorption site of CO^*^ by in situ DRIFTS (Figure S32, Supporting Information). The consistent CO^*^ band on Ru/ZrO_2_ and RuCo/ZrO_2_ indicated that the CO^*^ adsorption (2045 and 1970 cm^−1^) was attributed to the adsorption of CO^*^ on Ru site on RuCo/ZrO_2_ (Figure 5b). The absence of CO^*^ band on Co/ZrO_2_ demonstrated that weak CO adsorption is insufficient for subsequent hydrogenation reactions and thus rapid desorption. The CO^*^ was adsorbed on Ru site and continued reacting with hydrogen to form CH_4_ on RuCo/ZrO_2_, which was consistent with the result of a high CH_4_ selectivity. In addition, the RuCo/ZrO_2_ catalyst was conducted by in situ DRIFTS during a temperature‐programmed CO_2_ methanation process without light irradiation (Figure S33, Supporting Information). The peak intensities (HCO_3_^*^, HCOO^*^, CO^*^, and CH_4_) exhibited a significant enhancement, suggesting that the introduction of light promotes the conversion of reaction intermediates and increases the rate of CO_2_ to CH_4_. The H_2_ reduction behavior of RuCo/ZrO_2_ was analyzed by the H_2_ temperature programmed reduction (H_2_‐TPR Figure 5c). Similar H_2_ consumption was observed between Co/ZrO_2_ and RuCo/ZrO_2_, and the H_2_ consumption of Ru/ZrO_2_ was relatively minor. This observation confirmed that the incorporation of Ru has a slight effect on the H_2_ dissociation.^[^ ^15^, ^40^ ^]^ The RuCo/ZrO_2_ exhibited optimal CO_2_ adsorption ability by determining the CO_2_ temperature‐programmed desorption (CO_2_‐TPD, Figure S34, Supporting Information), which could be attributed to the enhancement of basicity after RuCo loading on ZrO_2_ (Figure S35, Supporting Information).^[^ ^13^, ^41^ ^]^

The catalytic mechanism was illustrated by DFT calculations (Figure 5d). The optimized configurations of the involved intermediates are shown in Figures S36 and S37 (Supporting Information). The adsorption‐free energies (E_ads_) of the CO_2_^*^ (−2.26 eV), HCO_3_^*^ (−1.94 eV), and HCOO^*^ (−1.77 eV) intermediates on Co/ZrO_2_ were similar to those on RuCo/ZrO_2_ (−2.26 eV, −2.17 eV, and −1.85 eV). The calculated E_ads_ of CO^*^ on RuCo/ZrO_2_ (−1.41 eV) was larger than that on Co/ZrO_2_ (−0.61 eV), indicating that the desorption of CO^*^ from Co/ZrO_2_ to form CO was achieved easily. For RuCo/ZrO_2_, the adsorption of CO^*^ was stable and allowed further hydrogenation to form subsequent intermediates (e.g., CHO^*^, CH_2_O^*^, CH_2_^*^, CH_3_^*^, and CH_4_^*^) that ensure the production of CH_4_. Based on the above analysis results, the catalytic mechanism that Ru–Co tandem catalytic site of RuCo/ZrO_2_ tuning the catalytic performance of CO_2_ methanation was proposed (Figure 5e). The catalytic site of Co–Ru loaded on ZrO_2_ favored the reaction steps involved in promoting CO_2_ to CO and subsequently hydrogenating CO to CH_4_, respectively, thereby achieving an efficient conversion of CO_2_ to CH_4_.^[^ ^10^, ^42^ ^]^

## 3 Conclusion

In summary, the RuCo/ZrO_2_ tandem catalyst with photothermal confinement effect was designed and synthesized. The high photothermal temperature of 323.7 °C was achieved due to the confinement of photothermal energy from the RuCo bimetallic nanoparticles by the infrared insulation and low thermal conductivity of the ZrO_2_ FC. The RuCo/ZrO_2_ catalyst exhibited superior photothermal CO_2_ methanation performance, realizing high CH_4_ selectivity (99%) and rate (169.93 mmol g_cat_^−1^ h^−1^). We proposed a tandem catalytic mechanism in which the Co site transformed CO_2_ into CO^*^, while the Ru site facilitated the adsorption of CO^*^ and subsequent hydrogenation to CH_4_ through combined reactivity, in situ DRIFTS, and DFT investigations. The two‐site RuCo/ZrO_2_ tandem catalyst significantly enhances the catalytic activity and selectivity of CO_2_ methanation. Our study presents a novel perspective on the design principles of photothermal catalysts for CO_2_ methanation.

## Conflict of Interest

The authors declare no conflict of interest.

## Supporting information

Supporting Information

## Data Availability

The data that support the findings of this study are available from the corresponding author upon reasonable request.
