# Supplementary material for: RuCo/ZrO2 Tandem Catalysts with Photothermal Confinement Effect for Enhanced CO2 Methanation
Source: Adv Sci (Weinh). 2024 Jul 10;11(34):2406828. doi: 10.1002/advs.202406828 (PMC11425663; doi:10.1002/advs.202406828)
Supplement: Supplementary file 1 — Supporting Information [file ADVS-11-2406828-s001.docx]

**Supporting Information:**

**RuCo/ZrO_2_ Tandem Catalysts with Photothermal Confinement Effect for Enhanced CO_2_ Methanation**

*Fan Yang^#^, Xiaoyu Liu^#^, Chuanshun Xing, Zizheng Chen, Lili Zhao, Xingwu Liu, Wenqiang Gao^*^, Luyi Zhu^*^, Hong Liu, Weijia Zhou^*^*

F. Yang, X. Liu, C. Xing, Z. Chen, L. Zhao, W. Gao, H. Liu, W. Zhou

Institute for Advanced Interdisciplinary Research (iAIR), School of Chemistry and Chemical Engineering, University of Jinan, Jinan, 250022, P. R. China

E-mail: ifc_gaowq@ujn.edu.cn (W. Gao); ifc_zhouwj@ujn.edu.cn (W. Zhou)

X. Liu, L. Zhu, H. Liu

State Key Laboratory of Crystal Materials, Shandong University, Jinan, 250100, P. R. China

E-mail: [zhuly@sdu.edu.cn](mailto:zhuly@sdu.edu.cn) (L. Zhu)

X. Liu

Synfuels China Technology Co., Ltd., Leyuan Second South Street Yanqi Development Zone Huairou, Beijing 101407, P. R. China

# These authors contributed equally to this work.

**Table of contents**

Supplementary Figure S1-37

Supplementary Table S1-5

Supplementary References

**Experimental section**

**Chemicals:** Zirconia fiber cotton (ZrO_2_ FC) was self-made in laboratory. Cobalt(Ⅱ) chloride hexahydrate (CoCl_2_·6H_2_O) was purchased from Shanghai Wokai Biotechnology Co., Ltd.. Urea (CH_4_N_2_O) was purchased from Sinopharm Chemical Reagent Co., Ltd.. Ruthenium(Ⅲ) chloride (RuCl_3_·3H_2_O) was purchased from Sinopharm Chemical Reagent Co., Ltd.. Deionized water was supplied with a Barnstead Nanopure Water System (Smart2Pure 6UV, 18.2 MΩ cm). Carbon dioxide (CO_2_, purity 99.99%), Argon (Ar, purity 99.99%) was obtained from Jinan De Yuan Gases Co., Ltd., Hydrogen (H_2_) was supplied with a CEHL-500 Hydrogen generator from Beijing Zhongjiao Jinyuan Technology Co., Ltd..

**Catalysts synthesis**

**a) Preparation of the ZrO_2_ FC**

Firstly, 0.5 g Poly (ethylene oxide) electrolyte (PEO) was dissolved in 10.0 ml of absolute ethanol, and 10 g of polyacetylacetonatozirconium (PAZ) was suspended in 50.0 ml absolute ethanol. The zirconia fibers are prepared by the electrostatic spinning method. The spinning voltage is 20 kV, and the receiving distance is 15-30 cm. The precursor fibers are heat treated in the air to obtain high entropy fibers, with a temperature rise rate of 1℃/min and a holding time of 2h.^[1]^

**b) Preparation of catalyst for bimetallic Ru and Co both loading on the ZrO_2_ FC (RuCo/ZrO_2_)**

Firstly, CoCl_2_·6H_2_O (100 mg), urea (100 mg), RuCl_3_·3H_2_O (1 mg), and ZrO_2_ (100 mg) were dissolved in deionized water (15 mL) stirring for 30 min. The prepared suspension was transferred to a 25 mL Teflon-lined autoclave and reacted at 200°C for 12 h. After being washed with deionized water three times and subjected to freeze-drying in a vacuum freeze dryer at -60°C for 20 h. The RuCo/ZrO_2_ catalyst was then calcined at 600°C for 4 h with a heating rate of 5°C/min under a 10% H_2_/Ar flow to obtain it.

The RuCo/ZrO_2_ catalysts with different amounts of Ru were labeled RuCo/ZrO_2_-x (x is Ru content according to the ICP measured)

**c) Preparation of catalysts for single metal Co (Co/ZrO_2_) and Ru (Ru/ZrO_2_) loading on the ZrO_2_ FC respectively**

The control samples Co/ZrO_2_ and Ru/ZrO_2_ were synthesized by the same method without RuCl_3_·3H_2_O and CoCl_2_·6H_2_O, respectively.

**d) Preparation of catalyst of the RuCo (RuCo) without ZrO_2_ FC**

The control sample RuCo was synthesized by the same method without ZrO_2_ FC.

**Photothermal catalytic performance test**

**a) The photothermal catalytic performance test in the closed batch system**

The samples (20 mg) were put in the bottle of the quartz batch reactor with 200 mL volume, the quartz batch reactor was equipped with a mixing system to make sure the stoichiometric amounts of H_2_ and CO_2_ (4:1). A 300 W Xe lamp equipped with a plano-convex lens, which the diameter of the spot was 2.5 cm. By adjusting the different light intensities (1.16, 1.47, 1.62, 1.76, 1.83, 1.96 W/cm^2^ ) to carry out the photothermal catalytic performance of RuCo/ZrO_2_ in the closed batch system. The evolved gas was analyzed using a gas chromatograph (HuiFen Analytical Instrument) equipped with a flame ionization detector (FID) and a thermal conductivity detector (TCD) was used to detect the products.

**b) The thermal catalytic performance test in the closed batch system**

The thermal catalytic performance was tested in the quartz tube by tube furnace. The quartz tube was evacuated and filled with the stoichiometric amounts of H_2_ and CO_2_ (4:1). The constant temperature was controlled by the tube furnace. The evolved gas was analyzed using a gas chromatograph (HuiFen Analytical Instrument) equipped with a flame ionization detector (FID) and a thermal conductivity detector (TCD) was used to detect the products.

**c) The photothermal catalytic performance test in real outdoor sunlight**

The outdoor tests were similar to the photothermal catalysis test on August 7, 2023. The outdoor sunlight was the light source. The 200 mL closed quartz reactor and the solar concentrator were used to drive the photothermal reaction. The gas was collected and analyzed by the gas chromatograph (HuiFen-901 Analytical Instrument) equipped with a flame ionization detector (FID) and a thermal conductivity detector (TCD) was used to detect the products.

**d) The Calculation of photothermal performance**

The production rate (mmol/g_cat_/h) was calculated as

CH_4_ rate = $\frac{n{\mathrm{CH}_{4}}_{\mathrm{out}}}{m_{\mathrm{cat}}\times t}$

CO rate = $\frac{{{n\mathrm{CO}}_{2}}_{\mathrm{out}}}{m_{\mathrm{cat}}\times t}$

The CO_2_ conversion was calculated as

CO_2_ conversion (%) = $\frac{{{n\mathrm{CO}}_{2}}_{\mathrm{in}}-{{n\mathrm{CO}}_{2}}_{\mathrm{out}}}{{{n\mathrm{CO}}_{2}}_{\mathrm{in}}}\times100\%$

The selectivity of CH_4_ was calculated as

Selectivity of CH_4_ (%) = $\frac{n{\mathrm{CH}_{4}}_{\mathrm{out}}}{{n\mathrm{CO}}_{\mathrm{out}}+n{\mathrm{CH}_{4}}_{\mathrm{out}}}\times100\%$

Where ${{n\mathrm{CO}}_{2}}_{\mathrm{in}}\mathrm{and}{{n\mathrm{CO}}_{2}}_{\mathrm{out}}$ are the mole numbers of CO_2_ in the inlet and outlet; ${n\mathrm{CO}}_{\mathrm{out}} \mathrm{and} n{\mathrm{CH}_{4}}_{\mathrm{out}}$the mole numbers of CO and CH_4_ in the outlet.

**Characterization**

Phase compositions of the as-made materials were performed on a powder X-ray diffractometer (Cu Kα, λ=0.15406 nm, Thermo Fisher, ARL Equinox 3000X). Morphologies of the as-made materials were identified by a field emission scanning electron microscope (FESEM, MERLIN Compact, Carl Zeiss) and a transmission electron microscope (TEM, a JEM-2100F Field Emission Electron Microscope, JPN) at an acceleration voltage of 200 kV. The light intensities were detected by the laser power meter (LP100, Changchun New Industries Optoelectronics Technology Co., Ltd.) The photothermal temperature and the corresponding infrared thermal images were recorded by Fotric 222s#L28 and a thermocouple. The temperature of the catalyst was measured by inserting a thermocouple underneath the catalyst (around 1 mm). The UV-Vis-NIR spectra was performed by UH4150 (Hitachi High-Technologies Corporation). The emissivity was tested by an infrared emissivity tester (Thermal Scientific Nicolet iS 50). The thermal conductivities were measured by a high temperature thermal conductivity tester (ISO-22007.2-2008).

X-ray photoelectron spectroscopic (XPS) measurement was performed using a PHI X-tool instrument (Ulvac-Phi). A gas chromatograph (HF-910, HuiFen Analytical Instrument Co., Ltd.) equipped with a flame ionization detector (FID) and a thermal conductivity detector (TCD) was used to detect the photocatalytic products. TPD was tested with the AUTO CHEM 2920. X-ray absorption spectroscopy (XAS) analyses were performed with Si (111) crystal monochromators at the BL14W Beamline at the Shanghai Synchrotron Radiation Facility (SSRF) (Shanghai, China). Before the analysis at the beamline, samples were placed into aluminum sample holders and sealed using Kapton tape film. The XAFS spectra were recorded at room temperature using a 4-channel Silicon Drift Detector (SDD) Bruker 5040. Co K-edge extended X-ray absorption fine structure (EXAFS) spectra were recorded in transmission mode and Ru K-edge in fluorescence mode. XAS data were processed and analyzed using the Demeter software package.

**Numerical calculations**

The COMSOL Multiphysics software was used to simulate the electromagnetic field distribution and temperature distribution. This method involves the discretization of Maxwell’s equations in the space domain to simulate the electric field distributions at different positions. Typically, an NFO NP absorbs NIR light (wavelength: 500nm; power intensity: 1.83 W/cm^2^), triggering the increase of the localized electromagnetic field, concentrating light energy on the NP, and delivering light-to-heat transformation as heat is generated. The optical absorption and generated volume power intensity could be calculated by COMSOL Multiphysics software solutions and were subsequently used as heat input for the temperature simulation. The basic heat transfer equation is given below:

$$\rho C_{p}\frac{\partial T(x,t)}{\partial t}+\rho C_{p}u\nabla T(x,t)=\nabla\cdot(k\nabla T\left( x,t \right))+Q_{1}$$

where $x$ and $t$ are the space vector and time, respectively. Thermal conductivity ($k$), density ($\rho$) velocity vector ($u$), and heat capacity ($C_{p}$) of NP. $T\left( x,t \right)$ is the local temperature and $Q_{1}$ represents the thermal energy induced by photo-to-thermal conversion within NFO NPs.

**In situ diffuse reflectance infrared Fourier transform spectroscopy (in situ DRIFTS) analysis.**

In situ DRIFTS was performed using a Tensor 27 spectrometer (Bruker) equipped with a MCT detector, by recording 32 scans at a resolution of 4 cm^-1^. The background spectra were first collected in the Ar atmosphere and subtracted from the sample spectra. Subsequently, a gas mixture of 64% H_2_ and 16% CO_2_ with 20% Ar was introduced into the reaction cell (50 mL/min). The light was introduced by an optical fiber equipped with a 300 W Xe lamp into the reaction cell passing through the quartz window. During the CO_2_ methanation, the spectra were collected under light irradiation and a temperature of 25–330°C with a heating rate of 10°C/min.

**Density functional theory (DFT) calculations**

First-principles calculations were carried out based on periodic DFT using a generalized gradient approximation within the Perdew-Burke-Ernzerh of exchange correction functional. The wave functions were constructed from the expansion of plane waves with an energy cutoff of 500 eV. Gamma centered k-point of 3×3×1 have been used for geometry optimization. The consistency tolerances for the geometry optimization are set as 1.0×10^-6^ eV/atom for total energy and 0.05 eV/Å for force, respectively. To avoid the interaction between the two surfaces, a large vacuum gap of 15 Å has been selected in the periodically repeated slabs. In free energy calculations, the entropic corrections and zero-point energy (ZPE) have been included. The free energy of species was calculated according to the standard formula:

∆G = E + ΔZPE + ΔH – ΔTS

where ZPE was the zero-point energy, ΔH was the integrated heat capacity, T was the temperature of the product, and S was the entropy.

**Supplementary Figure S1-36**


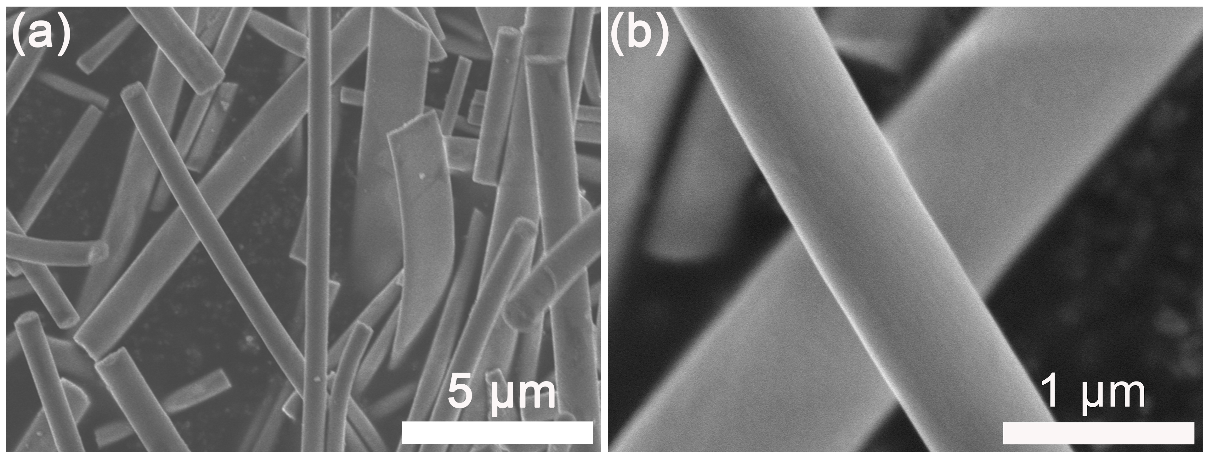


**Figure S1** SEM images of ZrO_2_.


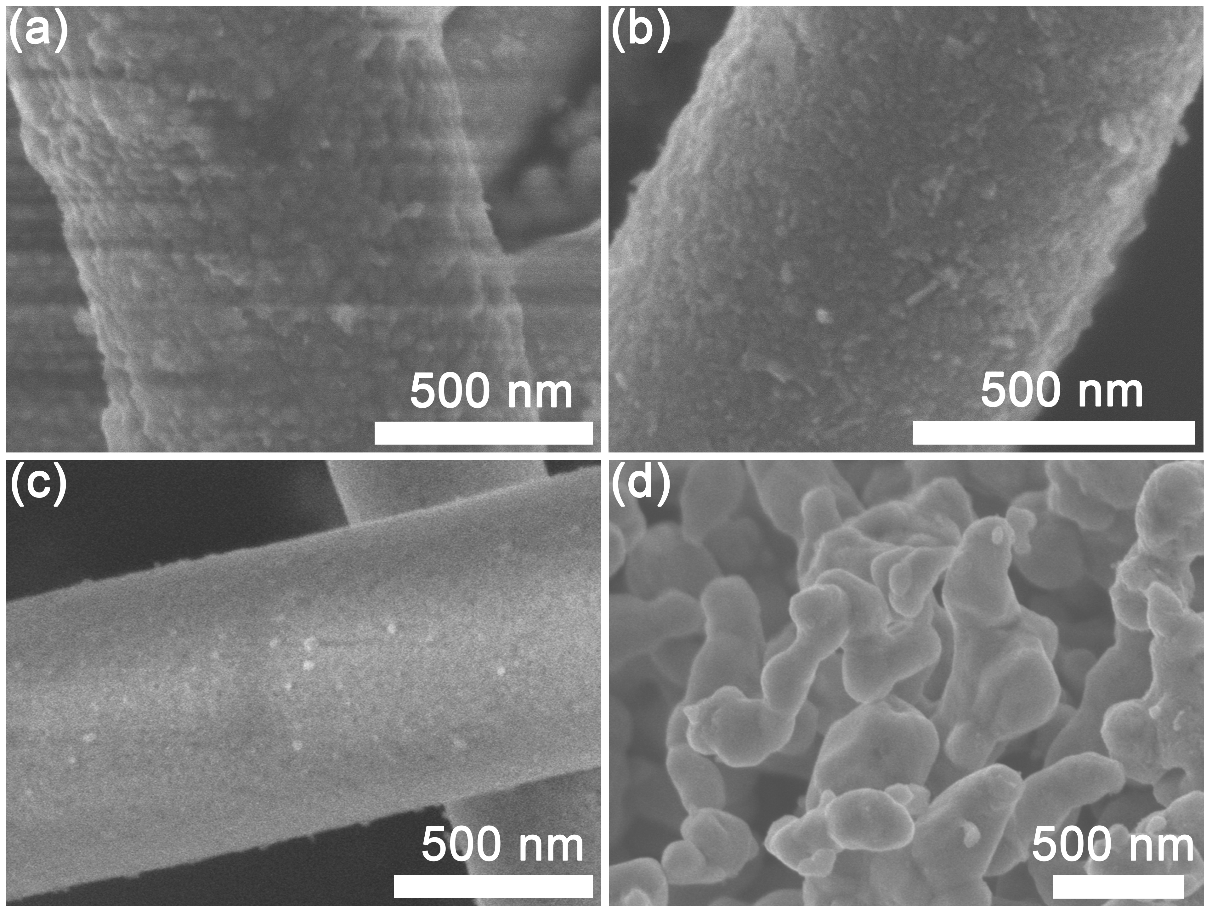


**Figure S2** SEM images of (a) RuCo/ZrO_2_, (b) Co/ZrO_2_, (c) Ru/ZrO_2_, and (d) RuCo.


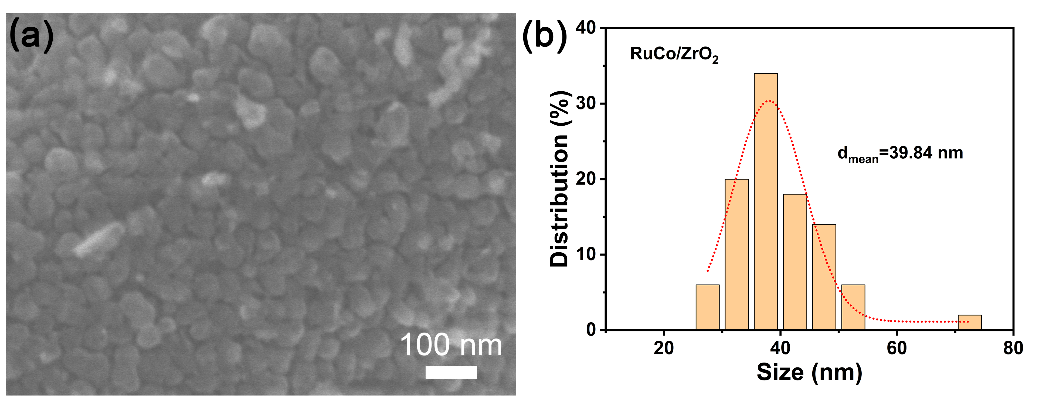


**Figure S3** (a) the SEM image of RuCo/ZrO_2_ and (b) the corresponding particle size distribution of RuCo nanoparticles.





**Figure S4** XRD patterns of ZrO_2_ and RuCo/ZrO_2_.

XRD patterns of ZrO_2_ and RuCo/ZrO_2_ showed the exposed diffraction peaks at 30.26°, 34.61°, 35.31°, 50.28°, 59.35°, 60.28°and 62.93° for (101), (002), (110), (112), (103), (211) and (202) of the tetragonal ZrO_2_ (PDF#79-1771).


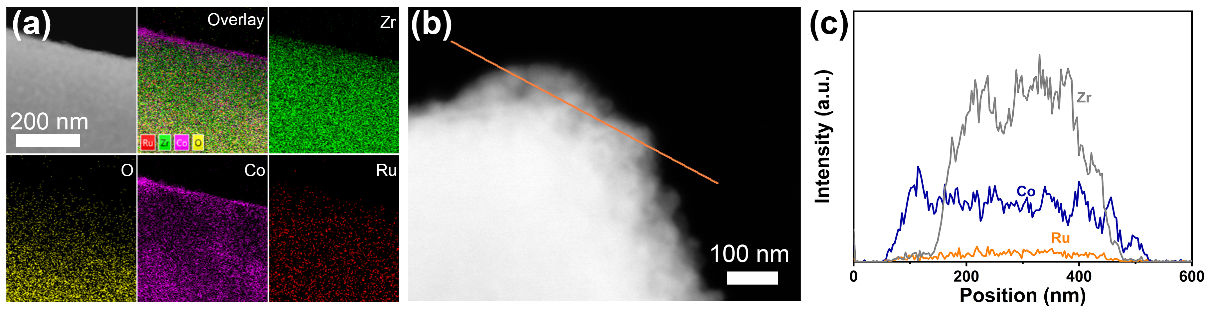


**Figure S5** (a) EDX mapping images of RuCo/ZrO_2_. (b, c) Line-scanning EDX of Ru, Co, and Zr elements for RuCo/ZrO_2_.


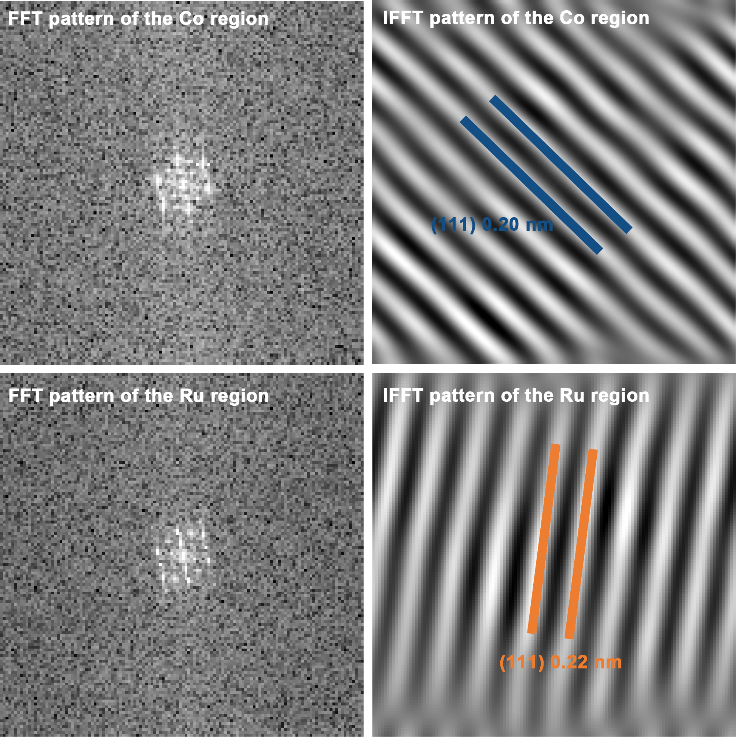


**Figure S6** FFT and IFFT patterns of the marked regions for RuCo/ZrO_2_.


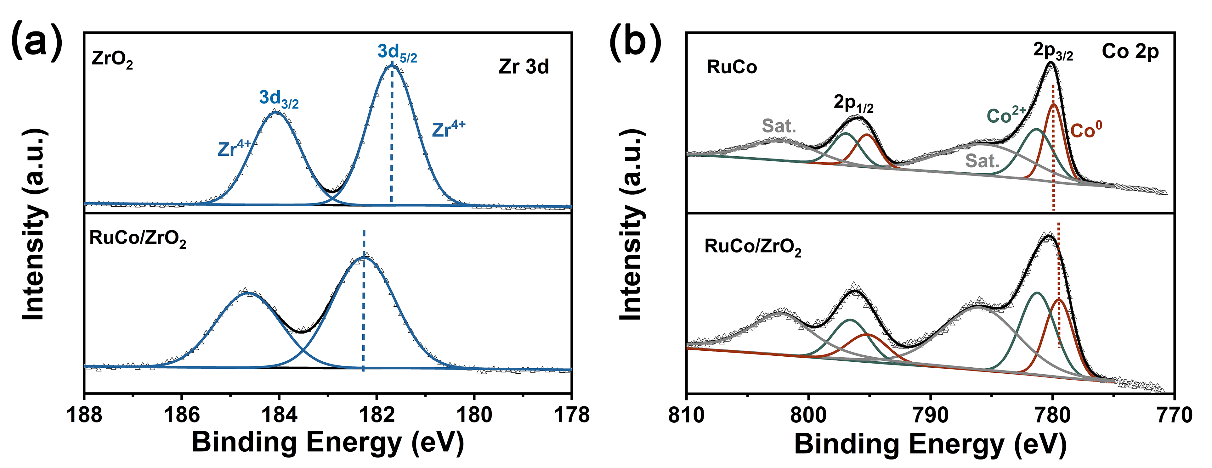


**Figure S7** (a) Zr 3d XPS spectra of ZrO_2_ and RuCo/ZrO_2_ (b) Co 2p XPS spectra of RuCo and RuCo/ZrO_2_.

A distinct pair of Zr^4+^ peaks at 184.07 eV and 181.69 eV were observed by XPS for ZrO_2_.^[2]^ The Co 2p peaks at 780.17 eV and 796.09 eV for RuCo and RuCo/ZrO_2_ were assigned to the Co 2p_3/2_ and Co 2p_1/2_, and the corresponding satellite peaks were observed at 802.64 eV and 785.21 eV, respectively.^[3]^ The Co 2p_3/2_ peak was deconvoluted into the signals of Co^0^ and Co^2+^.^[4]^ It could be rationalized that the presence of cobalt ion peaks is due to partial oxidation resulting from exposure to air.^[5]^


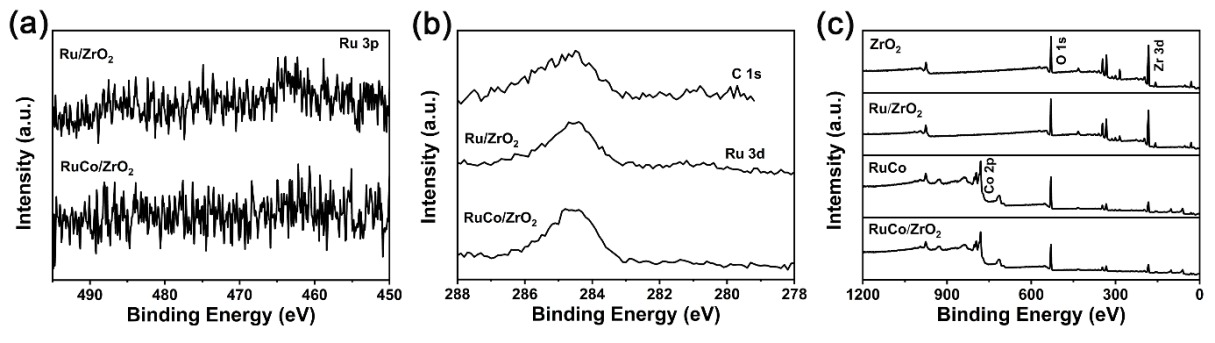


**Figure S8** (a) Ru 3d and (b) Ru 3p XPS spectra of Ru/ZrO_2_ and RuCo/ZrO_2_. (c) Full XPS spectra of the ZrO_2_, Ru/ZrO_2_, RuCo, and RuCo/ZrO_2_.





**Figure S9** XRD patterns of ZrO_2_, Ru/ZrO_2_, Co/ZrO_2_, and RuCo.





**Figure S10** UV-Vis-NIR absorption spectra of Co/ZrO_2_ and RuCo/ZrO_2_ with different Ru amounts.


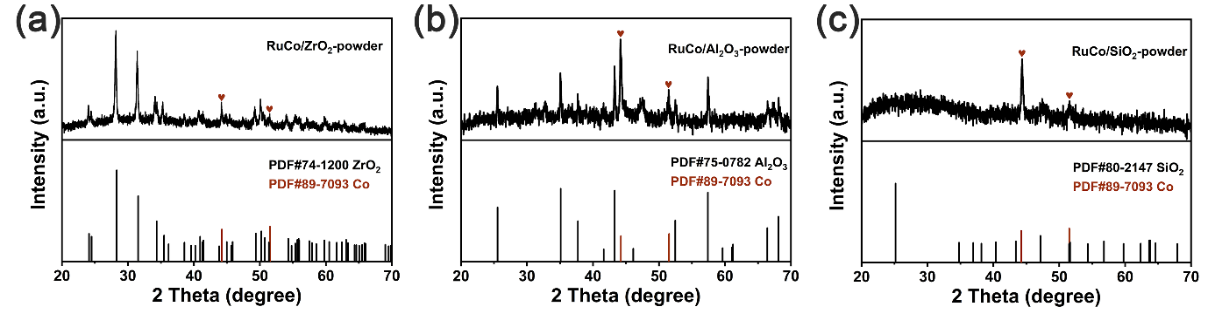


**Figure S11** XRD patterns of (a) RuCo/ZrO_2_-powder, (b) RuCo/Al_2_O_3_-powder, and (c) RuCo/SiO_2_-powder.


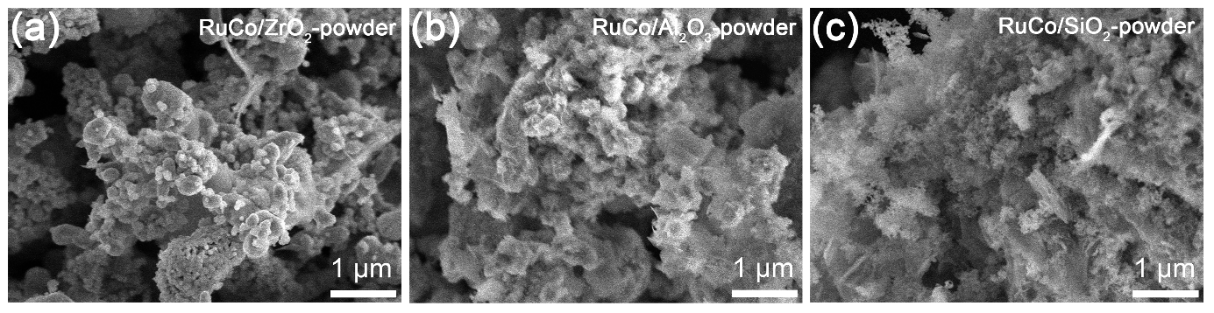


**Figure S12** SEM images of (a) RuCo/ZrO_2_-powder, (b) RuCo/Al_2_O_3_-powder, and (c) RuCo/SiO_2_-powder.





**Figure S13** The temperature rise curves of RuCo/ZrO_2_, RuCo/ZrO_2_-powder, RuCo/Al_2_O_3_-powder, and RuCo/SiO_2_-powder under the 1.83 W/cm^2^ irradiation.


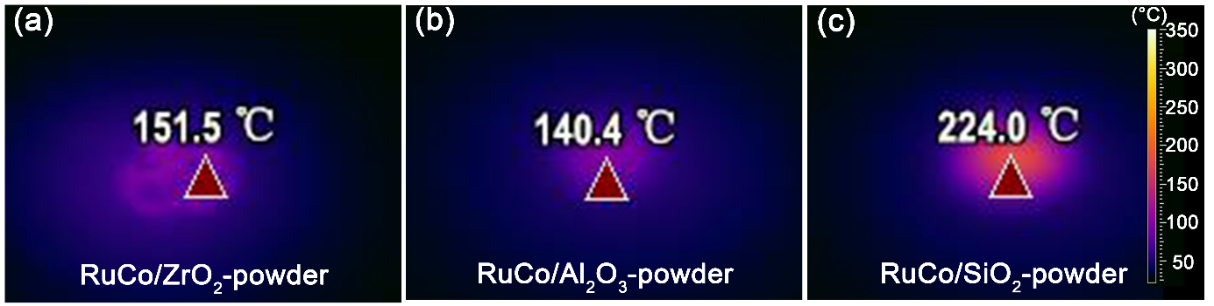


**Figure S14** The infrared thermal images of (a) RuCo/ZrO_2_-powder, (b) RuCo/Al_2_O_3_-powder, and (c) RuCo/SiO_2_-powder under the 1.83 W/cm^2^ irradiation.


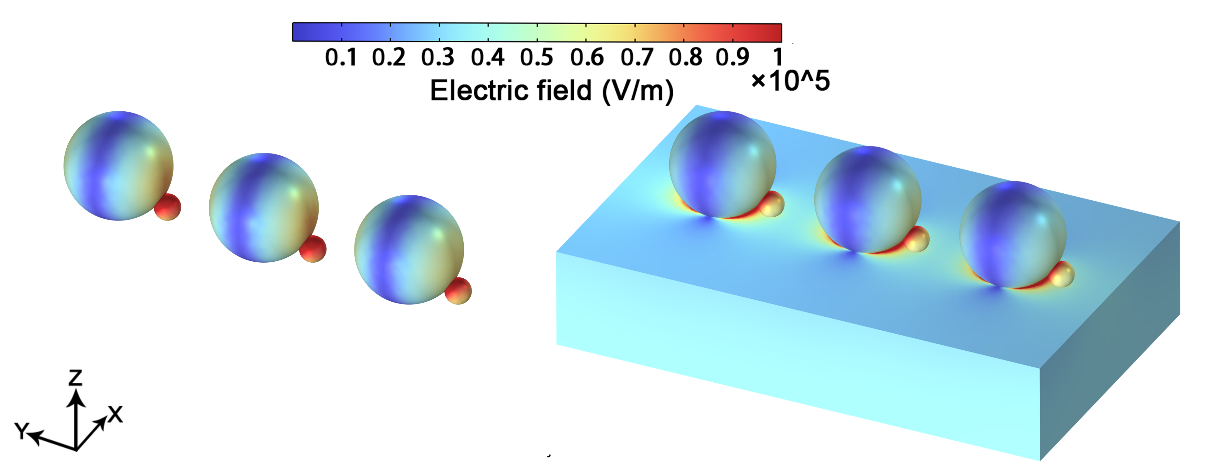


**Figure S15** Induced electric field distributions simulation on RuCo and RuCo/ZrO_2_ under light irradiation (500 nm, 1.83 W/cm^2^).


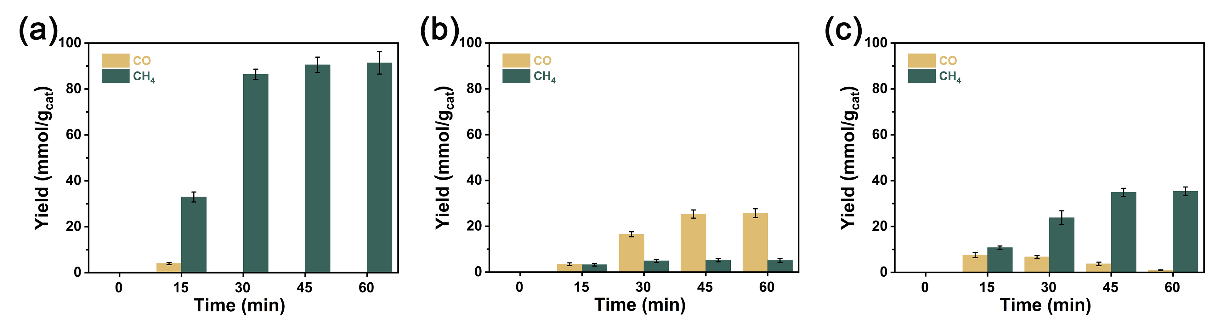


**Figure S16** The production yield of (a) RuCo/ZrO_2_, (b) Co/ZrO_2_, and (c) RuCo in 60 min.





**Figure S17** The production yield and CH_4_ selectivity of RuCo/ZrO_2_ (after 15 min of reaction), Co/ZrO_2_ ( after 45 min for Co/ZrO_2_), and RuCo (after 25 min for RuCo) at the 20% CO_2_ conversion.





**Figure S18** The production rate of CO and CH_4_ on the Ru/ZrO_2_ with different Ru amounts.





**Figure S19** The XRD patterns of RuCo/ZrO_2_ with different Ru amounts.


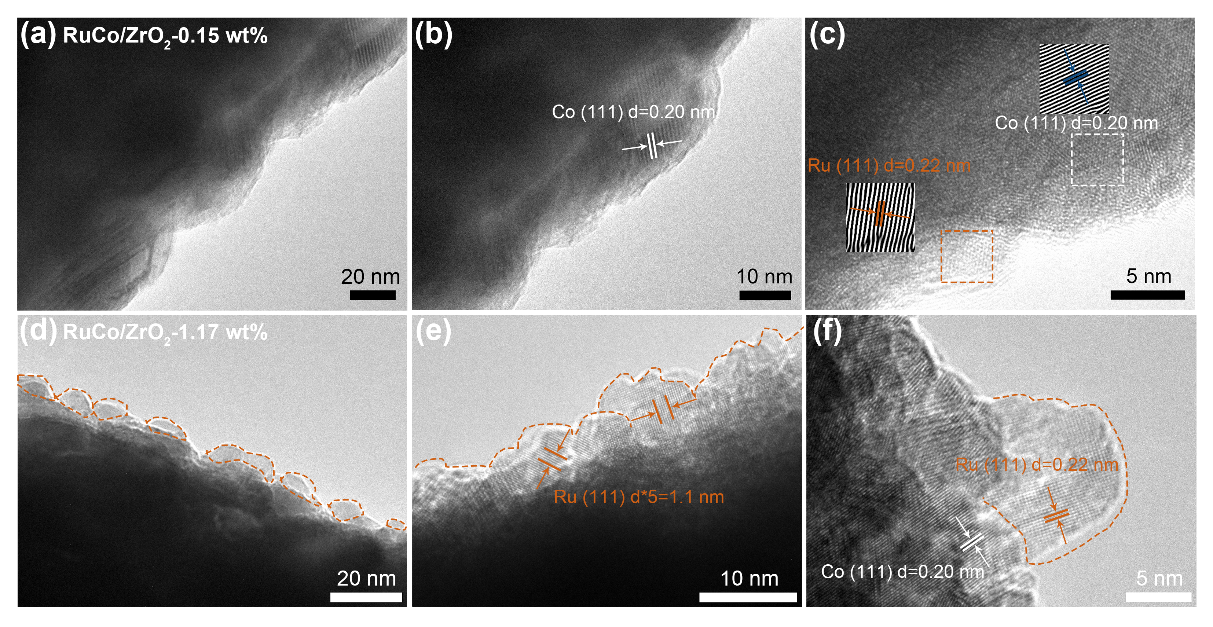


**Figure S20** TEM and HRTEM images of (a-c) RuCo/ZrO_2_-0.15 wt% and (d-f) RuCo/ZrO_2_-1.17 wt%.

**
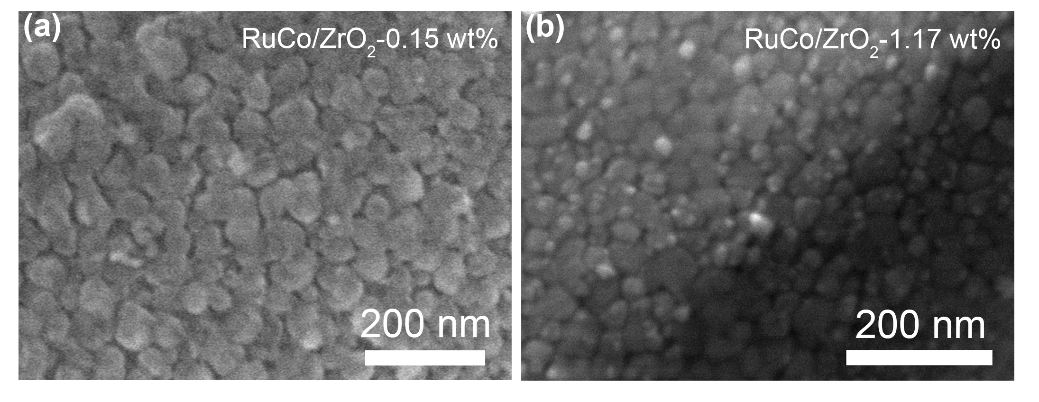
**

**Figure S21** SEM images of (a) RuCo/ZrO_2_-0.15 wt% and (b) RuCo/ZrO_2_-1.17 wt%.

**

**

**Figure S22** The production rate of RuCo/ZrO_2_ and M-RuCo/ZrO_2_.





**Figure S23** Cycle stability test of the RuCo/ZrO_2_ measured by the CO_2_ conversion.

**
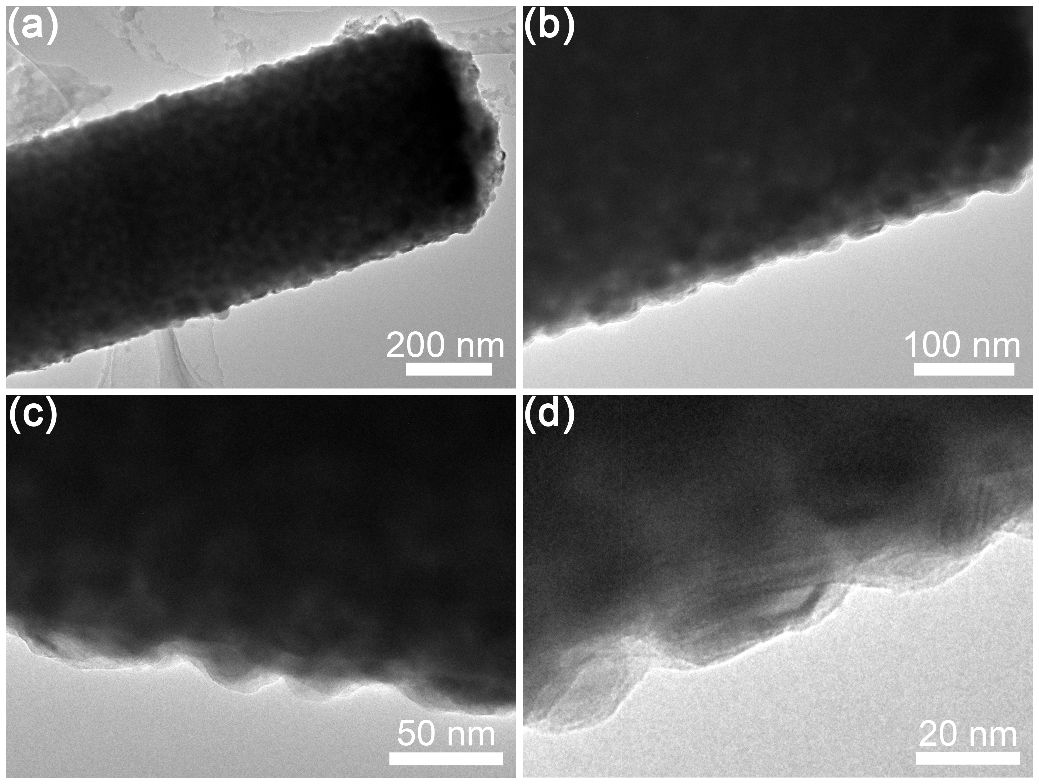
**

**Figure S24** The TEM images of RuCo/ZrO_2_ after the reaction.


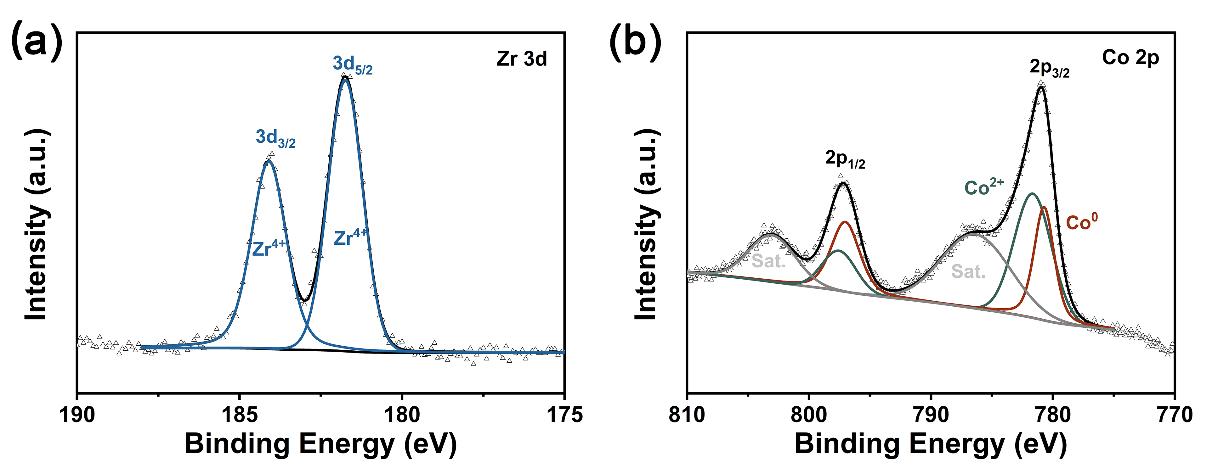


**Figure S25** The XPS spectra of RuCo/ZrO_2_ after the reaction.





**Figure S26** The XRD pattern of RuCo/ZrO_2_ after the reaction.





**Figure S27** The UV-Vis-IR absorption spectra of RuCo/ZrO_2_ before and after the reaction.





**Figure S28** Cycle stability test of the RuCo.


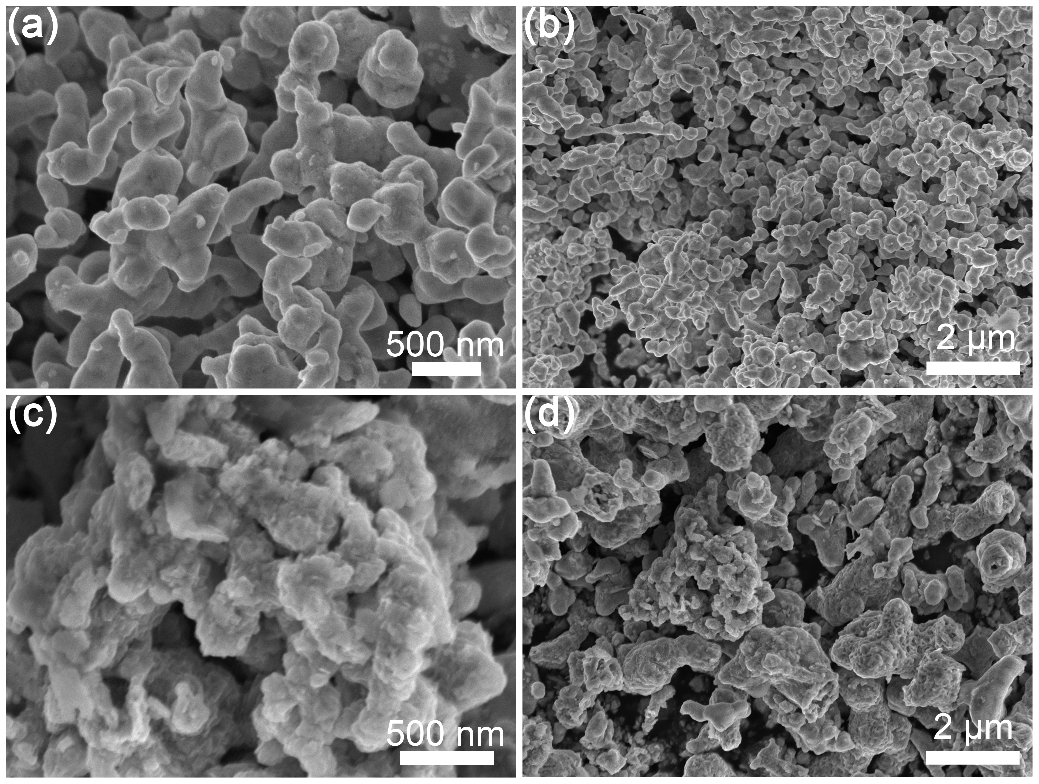


**Figure S29** The SEM images of (a, b) fresh and (c, d) used RuCo.


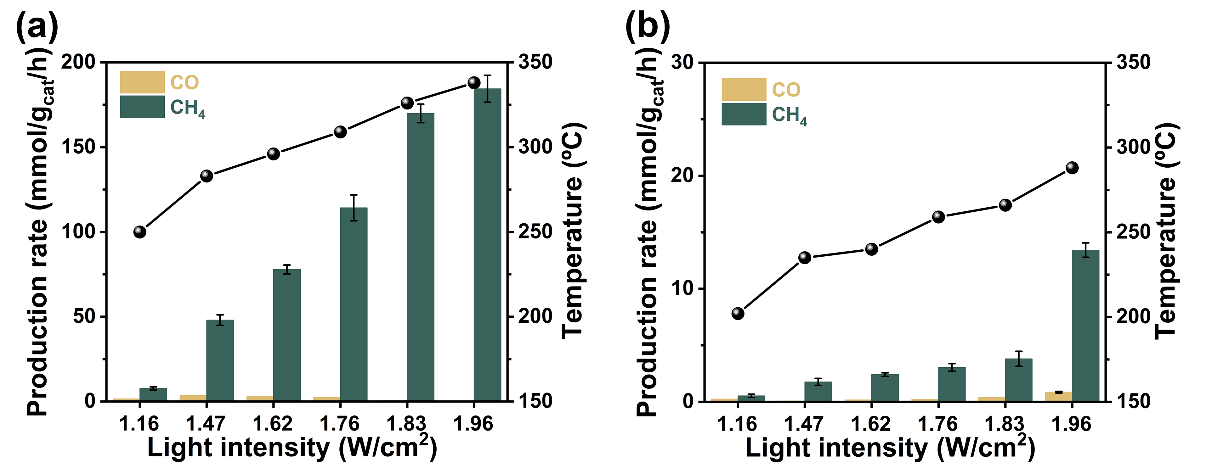


**Figure S30** Photothermal catalytic performance and photothermal temperature of the (a) RuCo/ZrO_2_ and (b) Ru/ZrO_2_ under different illumination conditions.


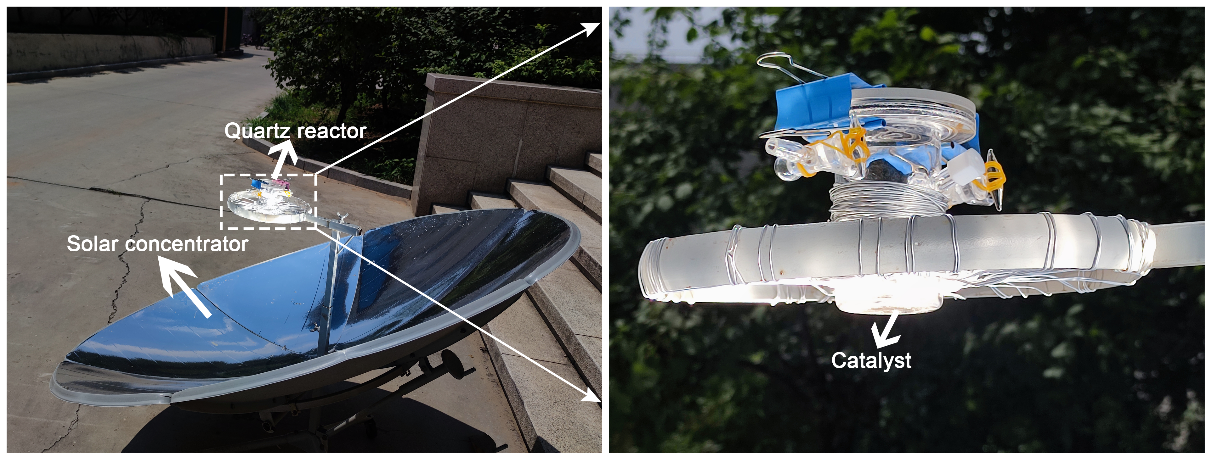


**Figure S31** Optical images of solar concentrator system.


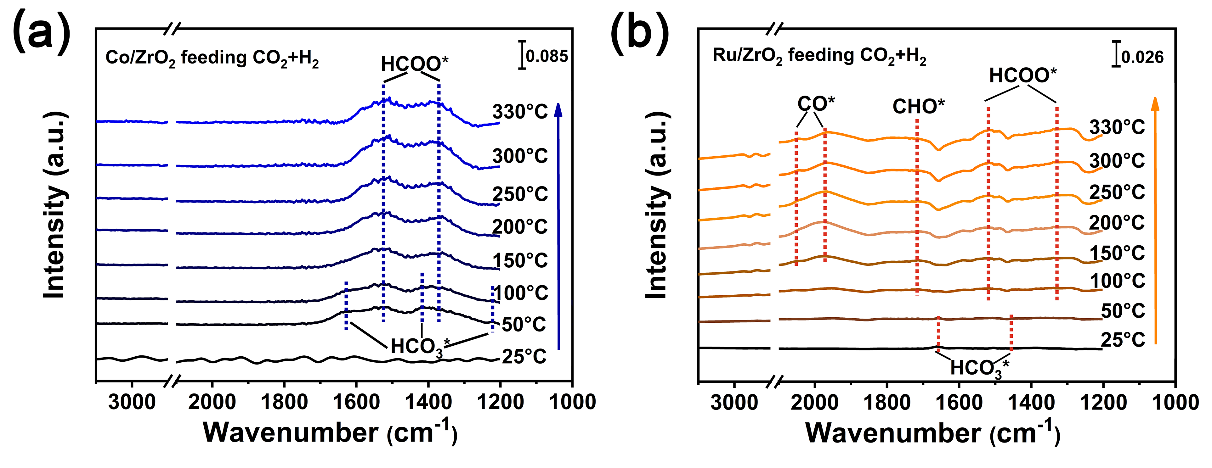


**Figure S32** In situ DRIFTS during both light irradiation and temperature-programmed CO_2_ methanation of a gas mixture containing the ratio of 1:4 with CO_2_/H_2_ on (a) Co/ZrO_2_ and (b) Ru/ZrO_2_.


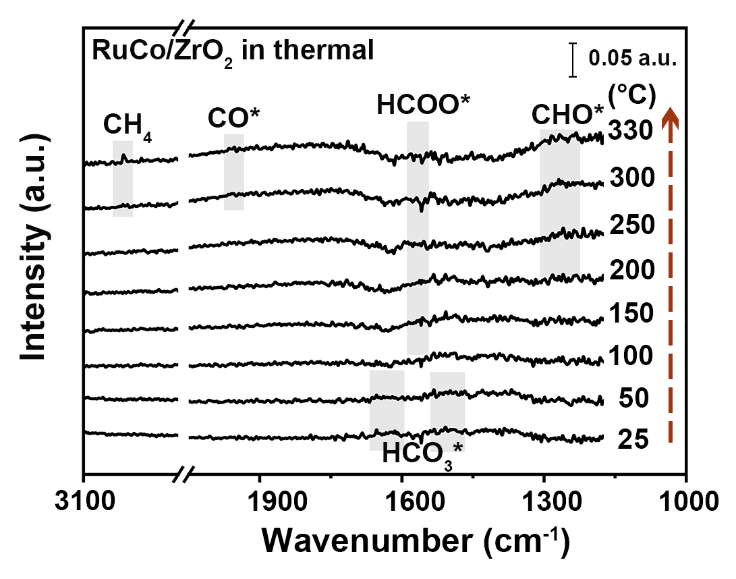


**Figure S33** In situ DRIFTS during temperature-programmed CO_2_ methanation of a gas mixture containing the ratio of 1:4 with CO_2_/H_2_ on RuCo/ZrO_2_.

**

**

**Figure S34** CO_2_-TPD comparison profiles of the Ru/ZrO_2_, Co/ZrO_2_, and RuCo/ZrO_2_.

**

**

**Figure S35** O 1s XPS spectra of ZrO_2_, Co/ZrO_2_, and RuCo/ZrO_2_.

The basicity related to the OH-groups of the catalyst surface, which were considered to facilitate the CO_2_ adsorption and activation.^[6]^ O_ads_ of the catalyst surface is directly related to the OH-groups.^[2]^ The increase in the ratio of O_ads_ suggests an enhancement of basicity.^[7]^ The ratio of O_ads_ in RuCo/ZrO_2_ was higher than that of Co/ZrO_2_ and ZrO_2_, as shown by the XPS results of O 1s. Therefore, the improved CO_2_ adsorption capacity of RuCo/ZrO_2_ is attributed to the enhancement of basicity after RuCo loading.

**
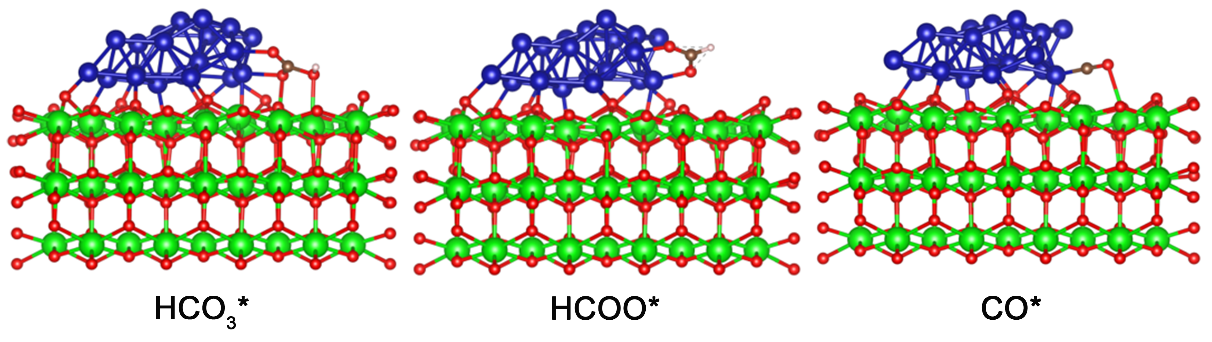
**

**Figure S36** The optimized geometric structure of involved intermediates of Co/ZrO_2_.

**
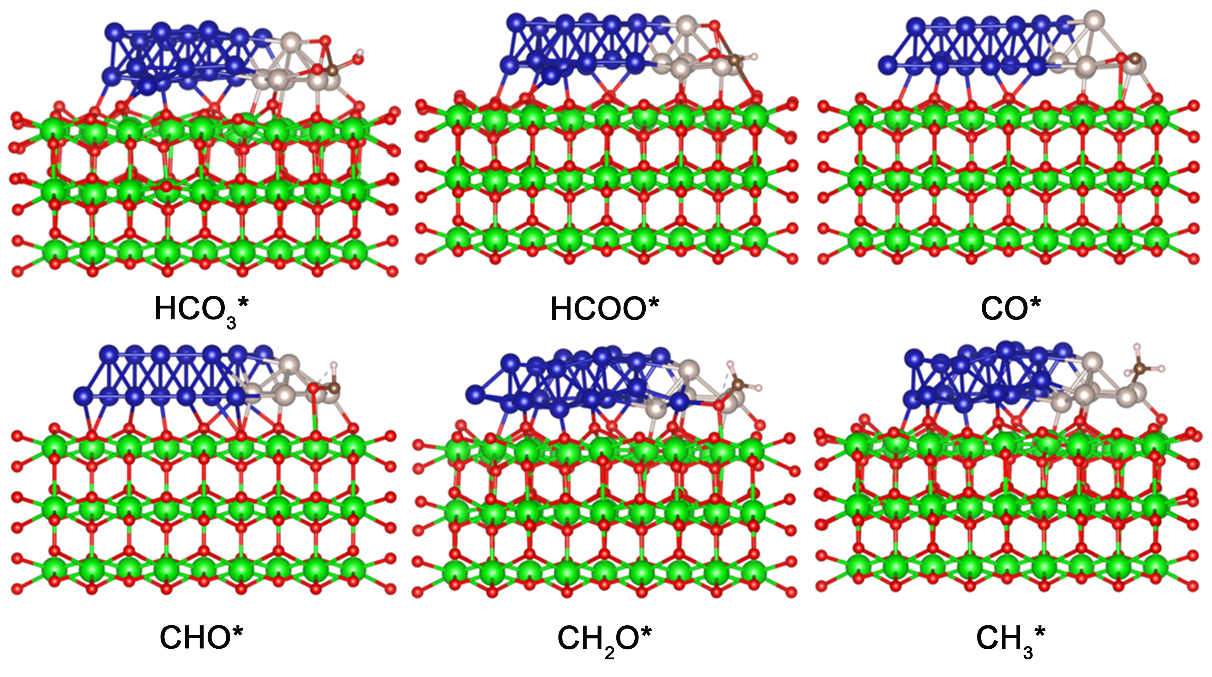
**

**Figure S37** The optimized geometric structure of involved intermediates of RuCo/ZrO_2_.

**Supplementary Table S1-5**

**Table S1** Metal content (Ru, Co) of all the samples

| **Sample** | **Ru (%)** | **Co (%)** |
| --- | --- | --- |
| Co/ZrO_2_ | 0 | 18.5000 |
| RuCo/ZrO_2_-0.15 wt% | 0.1546 | 18.3161 |
| RuCo/ZrO_2_-0.62 wt% | 0.6225 | 18.3341 |
| RuCo/ZrO_2_-0.94 wt% | 0.9353 | 18.9789 |
| RuCo/ZrO_2_-1.17 wt% | 1.1697 | 19.0661 |

**Table S2** Photothermal temperature of ZrO_2_, Ru/ZrO_2_, Co/ZrO_2,_ and RuCo/ZrO_2_ under the 1.83 W/cm^2^ irradiation monitoring by the thermocouple.

| **Sample** | **Photothermal temperature (°C)** |
| --- | --- |
| ZrO_2_ | 61.7 |
| Ru/ZrO_2_ | 198.3 |
| Co/ZrO_2_ | 249.6 |
| RuCo/ZrO_2_ | 298.8 |

**Table S3** Photothermal temperature of RuCo/ZrO_2_ under the same irradiation monitoring by the thermocouple and infrared camera respectively.

| **Irradiation(W/cm^2^)** | **Infrared camera (°C)** | **Thermocouple (°C)** |
| --- | --- | --- |
| 1.16 | 250 | 239 |
| 1.47 | 283 | 263 |
| 1.62 | 296 | 277 |
| 1.76 | 309 | 285 |
| 1.83 | 326 | 299 |
| 1.96 | 338 | 314 |

The temperature measured by a thermocouple is lower than that measured by an infrared camera due to a temperature gradient between the surface and the internal of the catalyst under light irradiation.^[8]^

**Table S4** The solar flux over time on August 7, 2023 from 8:30 to 17:30 in Jinan, Shandong China. The photothermal temperature of RuCo/ZrO_2_ achieved by the solar concentrator system.

| **Irradiation time of day** | **Solar flux**  **(kW/m^2^)** | **Photothermal**  **temperature (°C)** |
| --- | --- | --- |
| 8:30 | 0.39 | 308 |
| 9:30 | 0.59 | 352 |
| 10:30 | 0.72 | 367 |
| 11:30 | 0.87 | 365 |
| 12:30 | 0.92 | 380 |
| 13:30 | 0.91 | 376 |
| 14:30 | 0.93 | 363 |
| 15:30 | 0.87 | 353 |
| 16:30 | 0.54 | 317 |
| 17:30 | 0.25 | 210 |

**Table S5** The comparison of catalytic activity with other reported catalysts for photothermal catalytic CO_2_ conversion.

| Catalysts | Light source | Catalytic condition | Pressure (MPa) | Activity | Ref |
| --- | --- | --- | --- | --- | --- |
| RuCo/ZrO_2_ | 300 W Xe lamp (1.83 W/cm^2^) | without external heater | 0.1 | CH_4_ 168.27 mmol/g_cat_/h | This work |
| Co/Al_2_O_3_ | 300 W Xe lamp (1.3 W/cm^2^) | without external heater | 0.1 | CH_4_ 6.04 mmol/g_cat_/h | ^[9]^ |
| Co_15_HAP | 300 W Xe lamp (1.08 W/cm^2^) | 400°C | 0.1 | CO 62 mmol/g_cat_/h | ^[10]^ |
| Na-Co@C | 1000 W Xe lamp (19 kW/m^2^) | without external heater | 0.28 | CO_2_ ~9.5 mmol/g_cat_/h | ^[11]^ |
| Co@CoN&C | 300 W Xe lamp (light concentrator lens) | without external heater | 0.05 | CO 132 mmol/g_cat_/h | ^[12]^ |
| Co-PS@SiO_2_ | 300 W Xe lamp (20 Suns) | without external heater | 0.1 | CH_4_ 113.5 mmol/g_Co_/h | ^[13]^ |
| K^+^-Co-C | 300 W Xe lamp (2.8 W/cm^2^) | without external heater | 0.1 | CO 758 mmol/g_cat_/h | ^[14]^ |
| Cu/Pd/H_y_WO_3-x_ | 120 W Xe lamp (2.0 W/cm^2^) | 150°C | 0.1 | CO 1.18 mmol/g_cat_/h | ^[15]^ |
| Au@AuRu/g-C_3_N_4_ | 300 W Xe lamp (1.1 W/cm^2^) | 150°C | 1 | CH_4_ 0.103 mmol/g_cat_/h | ^[16]^ |
| Ag_24_Au/meso-Co_3_O_4_ | 300 W Xe lamp (0.187 W/cm^2^) | 240°C | 1.5 | CH_4_ 23 mmol/g_cat_/h | ^[17]^ |
| Pd_4_Ni_1_-SiO_2_ | 300 W Xe lamp (2.0 W/cm^2^) | without external heater | 0.1 | CO 125.5 mmol/g_cat_/h | ^[18]^ |
| ASA-c-Ag_8_Cu_1_ 3nm | 300 W Xe lamp (3.5 W/cm^2^) | without external heater | 0.1 | CH_4_ 5.4 mmol/g_cat_/h | ^[19]^ |
| Rh/Al | 300 W Xe lamp (5.9 W/cm^2^) | without external heater | 1.5 | CH_4_ 15 mmol/g_cat_/h | ^[20]^ |
| Co_7_Cu_1_Mn_1_O_x_ | 300 W Xe lamp (0.23 W/cm^2^) | 200°C | 0.1 | CH_4_ 14.5 mmol/g_cat_/h | ^[21]^ |
| Ir-CoO/Al_2_O_3_ | 300 W Xe lamp (2 W/cm^2^) | 250°C | 0.1 | CH_4_ 128.9 mmol/g_cat_/h | ^[22]^ |
| Ru-Au-S | 300 W Xe lamp (0.1 W/cm^2^) | 271.6°C | 1.3 | CH_4_ 47.2 mmol/g_Ru_/h | ^[23]^ |

**References**

[1] H. Y. Liu, X. Q. Hou, X. Q. Wang, Y. L. Wang, D. Xu, C. Wang, W. Du, M. K. Lü, D. R. Yuan, *J. Am. Chem. Soc*. **2004**, *87*, 2237.

[2] N. H. M. D. Dostagir, R. Rattanawan, M. Gao, J. Ota, J.-y. Hasegawa, K. Asakura, A. Fukouka, A. Shrotri, *ACS Catal.* **2021**, *11*, 9450.

[3] T. Y. Ma, S. Dai, M. Jaroniec, S. Z. Qiao, *J. Am. Chem. Soc.* **2014**, *136*, 13925.

[4] Z. Liu, X. Gao, B. Liu, W. Song, Q. Ma, T.-s. Zhao, X. Wang, J. W. Bae, X. Zhang, J. Zhang, *Appl. Catal. B Environ.* **2022**, *310*, 121303.

[5] Z. Liu, L. Zeng, J. Yu, L. Yang, J. Zhang, X. Zhang, F. Han, L. Zhao, X. Li, H. Liu, W. Zhou, *Nano Energy* **2021**, *85*, 105940.

[6] H. Wang, Q. Li, J. Chen, J. Chen, H. Jia, *Adv. Sci.* **2023**, *10*, 2304406.

[7] Z.-Y. Zhang, T. Li, X.-L. Sun, D.-C. Luo, J.-L. Yao, G.-D. Yang, T. Xie, *J. Catal.* **2024**, *430*, 115303.

[8] H. Jiang, L. Wang, H. Kaneko, R. Gu, G. Su, L. Li, J. Zhang, H. Song, F. Zhu, A. Yamaguchi, J. Xu, F. Liu, M. Miyauchi, W. Ding, M. Zhong, *Nat. Catal.* **2023**, *6*, 519.

[9] X. Chen, Q. Li, M. Zhang, J. Li, S. Cai, J. Chen, H. Jia, *ACS Appl. Mater. Interfaces* **2020**, *12*, 39304.

[10] Y. Peng, H. Szalad, P. Nikacevic, G. Gorni, S. Goberna, L. Simonelli, J. Albero, N. López, H. García, *Appl. Catal. B Environ.* **2023**, *333*, 122790.

[11] L. Liu, A. V. Puga, J. Cored, P. Concepción, V. Pérez-Dieste, H. García, A. Corma, *Appl. Catal. B Environ.* **2018**, *235*, 186.

[12] S. Ning, H. Xu, Y. Qi, L. Song, Q. Zhang, S. Ouyang, J. Ye, *ACS Catal.* **2020**, *10*, 4726.

[13] K. Feng, S. Wang, D. Zhang, L. Wang, Y. Yu, K. Feng, Z. Li, Z. Zhu, C. Li, M. Cai, Z. Wu, N. Kong, B. Yan, J. Zhong, X. Zhang, G. A. Ozin, L. He, *Adv. Mater.* **2020**, *32*, 2000014.

[14] H. Wang, S. Fu, B. Shang, S. Jeon, Y. Zhong, N. J. Harmon, C. Choi, E. A. Stach, H. Wang, *Angew. Chemie Int. Ed.* **2023**, *62*, e202305251.

[15] Y. F. Li, W. Lu, K. Chen, P. Duchesne, A. Jelle, M. Xia, T. E. Wood, U. Ulmer, G. A. Ozin, *J. Am. Chem. Soc.* **2019**, *141*, 14991.

[16] X. Zhang, H. Liu, Y. Wang, S. Yang, Q. Chen, Z. Zhao, Y. Yang, Q. Kuang, Z. Xie, *Chem. Eng. J.* **2022**, *443*, 136482.

[17] Y. Xiong, X. Liu, Y. Hu, D. Gu, M. Jiang, Z. Tie, Z. Jin, *Nano Res.* **2022**, *15*, 4965.

[18] Z. Zhu, X. Hu, X. An, M. Xiao, L. Zhang, C. Li, L. He, *Chem Asian J.* **2022**, *17*, e202200993.

[19] T. Shao, X. Wang, H. Dong, S. Liu, D. Duan, Y. Li, P. Song, H. Jiang, Z. Hou, C. Gao, Y. Xiong, *Adv. Mater.* **2022**, *34*, 2202367.

[20] G. Fu, M. Jiang, J. Liu, K. Zhang, Y. Hu, Y. Xiong, A. Tao, Z. Tie, Z. Jin, *Nano Lett.* **2021**, *21*, 8824.

[21] Z.-H. He, Z.-H. Li, Z.-Y. Wang, K. Wang, Y.-C. Sun, S.-W. Wang, W.-T. Wang, Y. Yang, Z.-T. Liu, *Green Chem.* **2021**, *23*, 5775.

[22] Y. Tang, T. Zhao, H. Han, Z. Yang, J. Liu, X. Wen, F. Wang, *Adv. Sci.* **2023**, *10*, 2300122.

[23] D. Mateo, D. De Masi, J. Albero, L. M. Lacroix, P. F. Fazzini, B. Chaudret, H. Garcia, *Chem. Eur. J.* **2018**, *24*, 18436.
